# From Explant Selection to Bioreactor Cultivation: Development of a *Hippeastrum × hybridum* Cell Culture Platform for Amaryllidaceae Alkaloid-Related Metabolism

**DOI:** 10.3390/plants15193041

**Published:** 2026-10-04

**Authors:** María Lenny Rodríguez-Escobar, Laura Torras-Claveria, Diego Hidalgo-Martínez

**Affiliations:** 1 Department of Biology, Healthcare and the Environment, Faculty of Pharmacy and Food Sciences, University of Barcelona, 08028 Barcelona, Spain; m-rodriguez@ub.edu (M.L.R.-E.); lauratorrascl@ub.edu (L.T.-C.); 2 Maria de Maeztu Unit of Excellence, Institute of Nutrition and Food Safety, University of Barcelona (INSA-UB), 08921 Santa Coloma de Gramenet, Spain

**Keywords:** *Hippeastrum × hybridum*, Amaryllidaceae alkaloids, plant cell suspension, bioreactor, coronatine, specialized metabolism, plant biotechnology

## Abstract

Amaryllidaceae alkaloids are pharmaceutically relevant specialized metabolites whose variable accumulation in planta has driven the development of controlled biotechnological production systems. This study developed an integrated *Hippeastrum × hybridum* cell-culture platform encompassing explant selection, callus induction, suspension culture establishment, bioreactor cultivation, and elicitation. Different explants were evaluated for callogenic and morphogenic competence, selected callus lines were adapted to suspension culture. B1- and BS-derived cultures were progressively expanded and transferred to 2 L bioreactors as an intermediate scale-up step and subsequently exposed to 1 µM coronatine (COR). Alkaloid extracts from cellular and extracellular fractions, together with in vitro and *ex vitro* plant tissues, were analyzed by GC–MS. Explant origin strongly influenced developmental responses, and B1- and BS-derived lines were selected based on sustained callus proliferation, biomass availability, suitable friability, and successful adaptation to liquid culture. Both lines maintained growth after transfer to 2 L bioreactors; in the bioreactor cultures evaluated, B1 showed greater estimated net biomass accumulation than BS. Their alkaloid-related profiles differed markedly between lines and sampling times during the elicitation phase. Notably, an extracellular signal assigned to *O*-methylnorbelladine was detected in B1 at D7, consistent with retention of metabolic competence related to Amaryllidaceae alkaloid biosynthesis. The developed system should therefore be regarded as a proof-of-concept laboratory-scale platform rather than an optimized alkaloid-production process, providing a basis for future studies with biological replication, quantitative process optimization, and application to *Hippeastrum* species or genotypes preselected for pharmaceutically relevant alkaloid profiles.

## 1. Introduction

The Amaryllidaceae family is an important source of structurally diverse and pharmacologically relevant alkaloids. Among these compounds, galantamine is a clinically established example because of its use in the symptomatic treatment of Alzheimer’s disease, while the biological activities and pharmacological potential of many other Amaryllidaceae alkaloids remain under investigation [1,2]. However, their often limited and variable accumulation in planta, influenced by genetic, developmental, and environmental factors, constrains their reliable pharmaceutical exploitation [3,4]. Plant *in vitro* systems, particularly cell suspension cultures, offer an alternative for producing reproducible biomass under controlled conditions and can be progressively scaled into biotechnological production platforms [5,6].

Within this family, *Hippeastrum* is particularly relevant because of its pronounced phytochemical diversity and the broad biological potential reported for its alkaloids. Previous surveys have demonstrated substantial interspecific and intraspecific variability within the genus, including compounds belonging to the galanthamine-, lycorine-, homolycorine-, crinine/haemanthamine-, and other structural groups [7,8,9]. In a recent study of 27 samples representing 23 Bolivian *Hippeastrum* species, 48 Amaryllidaceae alkaloids were identified, with some species showing notable amounts of galanthamine-type compounds whereas others were characterized by lycorine- or homolycorine-rich profiles and distinct cholinesterase-inhibitory activities [8]. Thus, the pharmaceutical interest of *Hippeastrum* is not restricted to galanthamine production but also resides in the chemical and biological diversity occurring across species and genotypes.

The present study did not aim to select a genotype with maximal galanthamine accumulation. Instead, *Hippeastrum × hybridum* was used as a readily available horticultural model to establish the successive experimental stages required for a cell-culture platform. The availability of a complete adult plant allowed multiple organs to be destructively sampled as explant sources and provided sufficient material for iterative development of callus, suspension, and scale-up procedures. This experimental accessibility is relevant because wild *Hippeastrum* resources of phytochemical interest may be geographically restricted or endemic and therefore less suitable for extensive initial platform optimization [8]. The strategy adopted here was therefore to establish the biotechnological workflow first and subsequently consider its transfer to species or genotypes selected on the basis of a desired alkaloid profile. Accordingly, the analytical stage was not designed around predefined target metabolites; rather, GC–MS profiling was used to characterize the compounds that could be structurally assigned in the different plant tissues and cell-culture fractions.

Alkaloid accumulation is strongly influenced by tissue identity, with marked differences reported among leaves, bulbs, roots, and specific bulb regions of *Hippeastrum papilio* [10,11]. Explant origin also determines the developmental response under *in vitro* conditions. Regenerative responses have been reported from leaf bases, floral scapes, bulb scales, ovaries, and other inflorescence-derived tissues of *Hippeastrum* hybrids, whereas floral tepals have also been used for somatic embryogenesis [12,13,14]. Similar interactions between culture conditions, morphogenesis, and alkaloid accumulation occur in other Amaryllidaceae [15]. Thus, explant selection is relevant both for regeneration and for identifying tissues capable of sustained biomass proliferation.

The transition from callus to cell suspension is a critical step in developing a plant cell biofactory. Calli showing sustained proliferation and suitable friability are generally preferred for the establishment of cell suspension cultures, although the growth-regulator requirements of solid and liquid systems may differ [16]. In related Amaryllidaceae systems, alkaloid accumulation has also been shown to vary with the degree of tissue differentiation and culture conditions [17,18,19]. In *Leucojum aestivum*, callus cultures showed more limited alkaloid profiles than differentiated tissues [18], while precursor-feeding experiments demonstrated that *in vitro* Amaryllidaceae cultures can retain biosynthetic competence for alkaloid formation [20]. Once stable suspensions are established, bioreactor cultivation can improve process control and provide a route toward progressive scale-up, although performance may change with reactor geometry, aeration, mixing, oxygen transfer, and hydrodynamic conditions [21,22,23]. In *L. aestivum* cultivated in RITA^®^ temporary-immersion bioreactors, carbohydrate type and concentration affected biomass development, alkaloid diversity, and the accumulation of galantamine and lycorine, while conditions favouring maximal biomass did not necessarily coincide with those favouring alkaloid accumulation [24].

Elicitation can subsequently be used to stimulate specialized metabolism. Coronatine (COR), a jasmonate-related elicitor, has been shown to enhance specialized metabolite production in several plant cell culture systems. In *Taxus media*, COR increased taxane accumulation and showed greater elicitation activity than methyl jasmonate [25], while later studies confirmed its effects on paclitaxel biosynthesis and metabolite distribution between cellular and extracellular fractions [26,27]. However, integrated *Hippeastrum* cell suspension systems combining line selection, bioreactor cultivation, and elicitation remain poorly developed.

In this context, the present study aimed to establish a proof-of-concept and progressively scalable *Hippeastrum × hybridum* cell-culture platform for the controlled cultivation and characterization of Amaryllidaceae alkaloid-related metabolism. Accordingly, *H. × hybridum* was used as a model for platform establishment rather than as a genotype preselected for high galanthamine production. Different explant types were first evaluated under contrasting auxin–cytokinin combinations to identify their callogenic and morphogenic responses. Callus lines combining sustained proliferation, sufficient biomass availability, morphological stability, and suitability for liquid culture were then used to establish cell suspensions, allowing differences between conditions favoring solid-phase callus growth and liquid-phase biomass proliferation to be identified. Selected B1- and BS-derived lines were subsequently expanded and scaled up to 2 L bioreactors, where their biomass accumulation and physiological behavior were characterized. Finally, the bioreactor-grown cultures were elicited with COR, and cellular and extracellular fractions collected over time were independently analyzed by GC–MS for comparative chemical profiling, without predefined target metabolites. Thus, the study follows the progressive development of cell biofactories from explant selection through suspension establishment to an intermediate 2 L bioreactor scale-up stage and provides a framework for the controlled biotechnological study and future investigation and optimization of Amaryllidaceae alkaloid-related metabolism in controlled cell-culture systems.

## 2. Results

### 2.1. In Vitro Establishment and Explant-Dependent Callogenic Response

**Figure 1:**
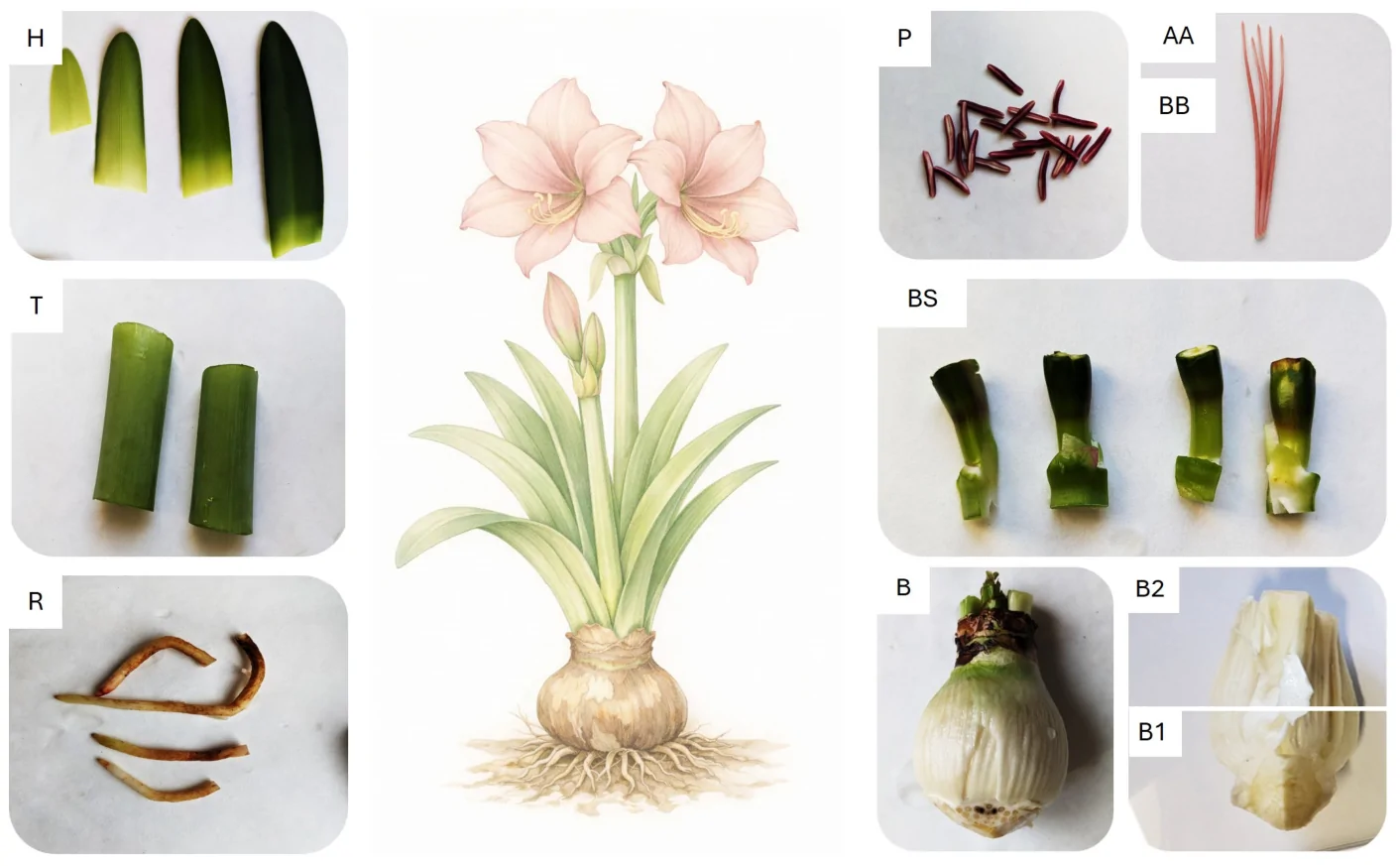
**Representative illustration of *Hippeastrum × hybridum* and original explant types used for *in vitro* culture establishment.** The central image is a representative schematic illustration of an adult *Hippeastrum × hybridum* plant and does not correspond to the actual donor plant. The surrounding photographs show the original organs or tissues used as experimental explants: leaf (H), floral scape (T), root (R), ovary (BS), isolated anthers (P), stamen filaments obtained from open flowers (AA), stamen filaments obtained from closed flower buds (BB), bulb (B), basal bulb fraction including the basal plate (B1), and apical bulb fraction without the basal plate (B2). The assigned codes were maintained throughout the study to ensure traceability of the plant material and derived lines. The figure is intended to document explant identity and origin rather than quantitative culture responses, which are summarized in Table 1.

The different anatomical regions of *Hippeastrum × hybridum* evaluated as explant sources are shown in Figure 1, whereas their quantitative callogenic and organogenic responses are summarized in Table 1. During the *in vitro* establishment of *Hippeastrum × hybridum*, visually viable tissue was retained in multiple experimental lines, although physiological responses varied among explant types. The basal and apical bulb fractions (B1 and B2, respectively) and root segments (R) showed high initial stability, whereas leaf (H), ovary (BS), and floral scape (T) explants displayed greater heterogeneity, mainly associated with differences in the progression of oxidation and necrosis. Oxidation did not necessarily result in an immediate loss of viability, as browned areas frequently coexisted with visually viable tissue. The detailed physiological behavior of the different explants during establishment is summarized in Table S1.

**Figure 2:**
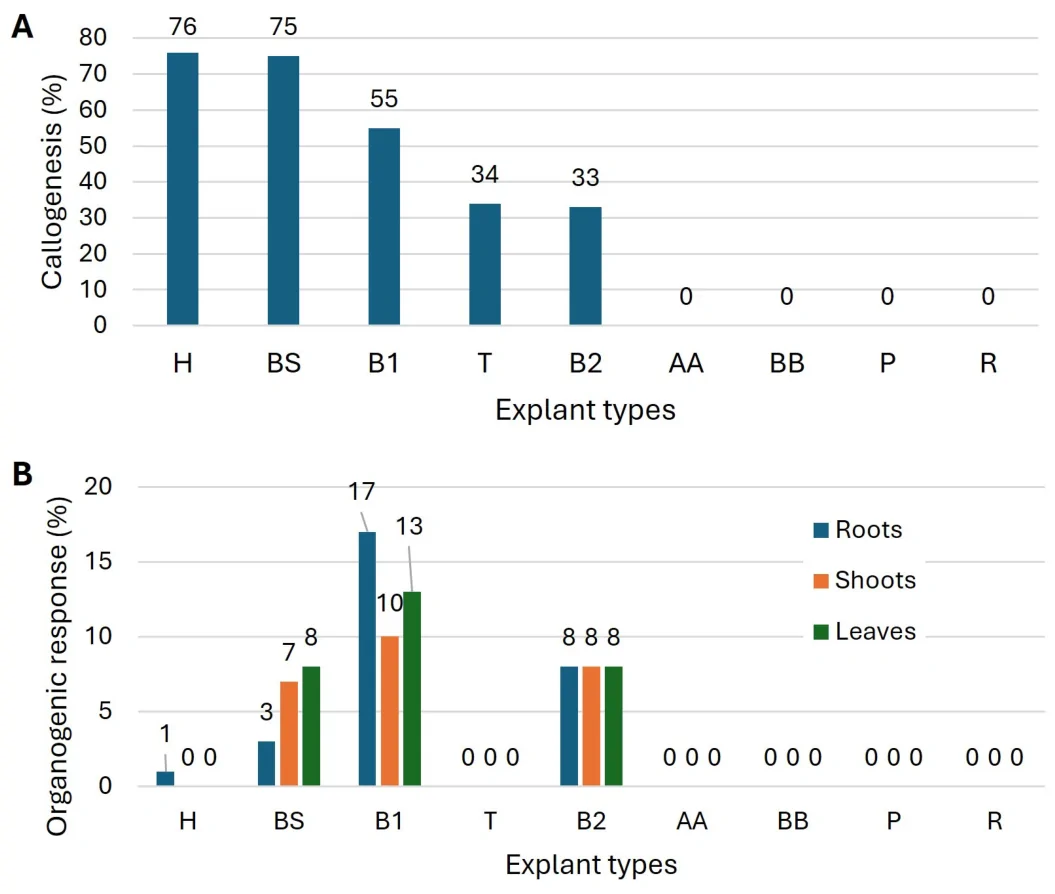
**Callogenic and organogenic responses of different *Hippeastrum × hybridum* explants during *in vitro* culture.** (**A**) Frequency of callus formation and (**B**) frequency of individual organogenic responses, including leaf (H), ovary (BS), basal bulb fraction including the basal plate (B1), floral scape (T), and apical bulb fraction without the basal plate (B2) explants. Response frequencies were calculated relative to the number of initial explants included in the corresponding dataset. Panel (**B**) shows the individual root, shoot, and leaf responses observed during the initial organogenic-response stage. Bulbiform structures developed subsequently during advanced regeneration and micropropagation and are therefore reported separately in Figure 4 and Table S3. Each explant was counted only once regardless of the number or type of structures produced. AA, BB, P, and R explants, which showed no callogenic or organogenic response, are reported in Table 1 and were omitted from the figure for clarity.

Callus induction showed a marked dependence on the anatomical origin of the explant (Figure 2A; Table 1). Leaf and ovary explants exhibited the highest callogenesis frequencies, reaching 76.3% (122/160) and 75.0% (90/120), respectively. The basal bulb fraction (B1) showed an intermediate response, with 55.0% (33/60) of the explants producing callus, whereas the floral scape and the apical bulb fraction (B2) displayed lower frequencies of 34.3% (34/99) and 32.5% (26/80), respectively. In contrast, stamen filaments obtained from open flowers (AA) and closed flower buds (BB), isolated anthers (P), and root segments did not develop callus masses during the evaluated period.

**Figure 3:**
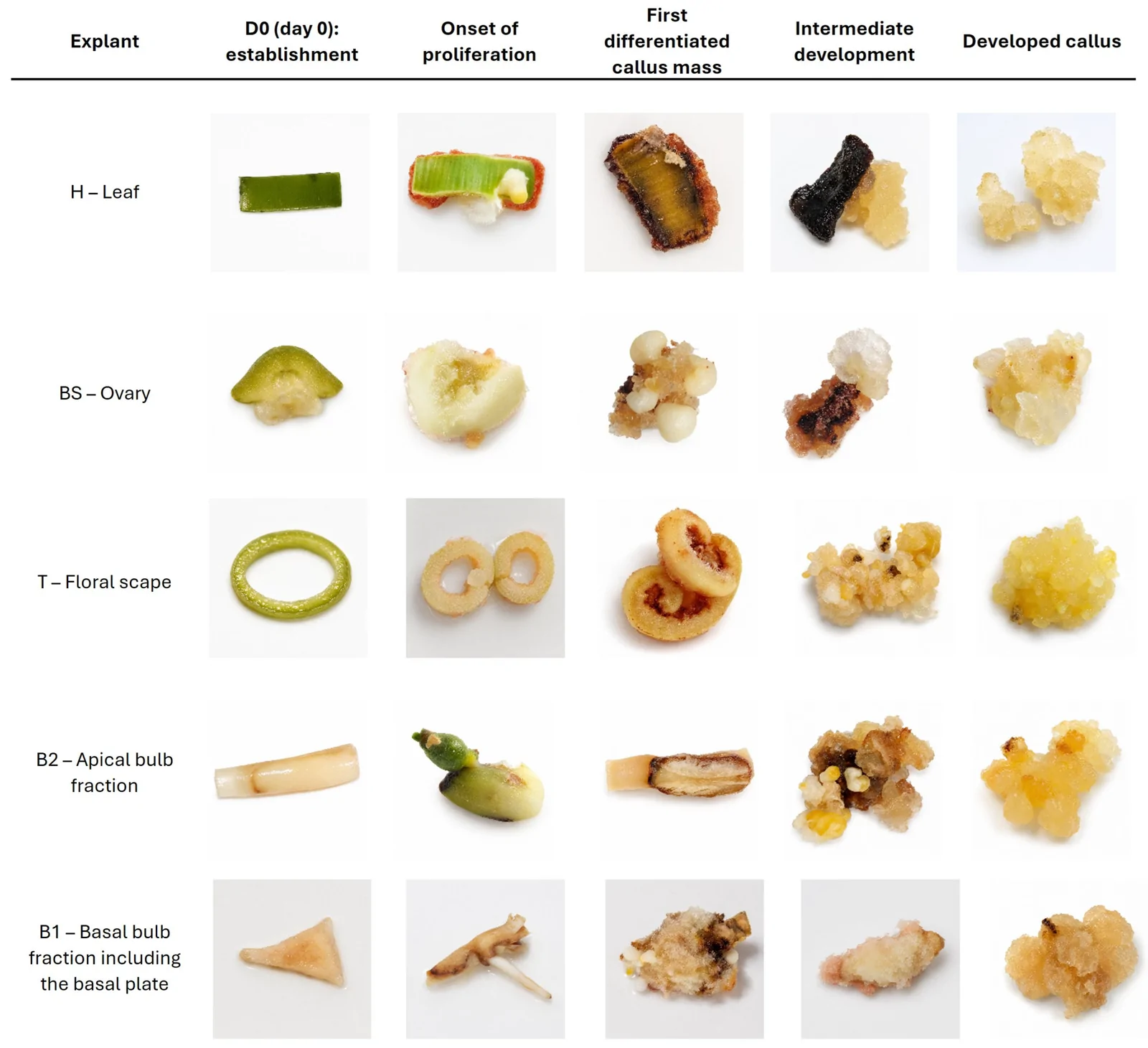
**Representative morphological progression of callus formation in responsive *Hippeastrum × hybridum* explants.** The stages represent a summarized morphological sequence rather than equivalent chronological sampling points across explant types or treatments. The latency of initial proliferation and the development of a clearly differentiated callus mass varied among explant types, treatments, and experimental lines. B1 showed the earliest proliferative response, while B2 also responded during the first weeks of culture, although callus consolidation varied among lines. H showed detectable proliferation from approximately W3, whereas clearly differentiated callus masses in BS and T became more evident around W8. Only explant types with confirmed callogenesis are shown. H, leaf; BS, ovary; T, floral scape; B2, apical bulb fraction; B1, basal bulb fraction including the basal plate.

The temporal dynamics of the response also varied among explants and treatments. The first proliferative changes were observed during the early weeks of culture in bulb and leaf tissues, whereas the establishment of clearly differentiated callus masses generally occurred at later stages. In H, proliferative growth became detectable from approximately week 3 (W3), although well-defined callus masses were more evident around W8. B1 showed detectable proliferation from approximately W2, with clearly differentiated callus observed from approximately W4, whereas in B2 callus consolidation occurred mainly between W5 and W8. In BS, proliferative changes preceded clearly differentiated callus formation, which became evident mainly around W8. In T, clearly differentiated callus was generally observed around W8, although a markedly delayed response occurred in T8 at approximately W24. The complete temporal dynamics of callus development are summarized in Table S2, while representative morphological progression of responsive explants is shown in Figure 3.

Callus induction frequency was not necessarily associated with the subsequent persistence or characteristics of the callus tissue. In H, some treatments induced early and abundant proliferation but were subsequently accompanied by increasing oxidation, whereas other lines retained proliferative tissue for longer periods. Clear differences were also observed among BS lines, ranging from abundant callus masses that later developed oxidation to nodular or granular tissues showing greater stability. In B1 and B2, some treatments produced persistent callus despite their lower overall induction frequencies compared with H and BS. These differences indicated that callogenesis frequency alone was insufficient to select material for subsequent biotechnological stages and supported the independent evaluation of line stability, proliferative capacity, and friability.

**Figure 4:**
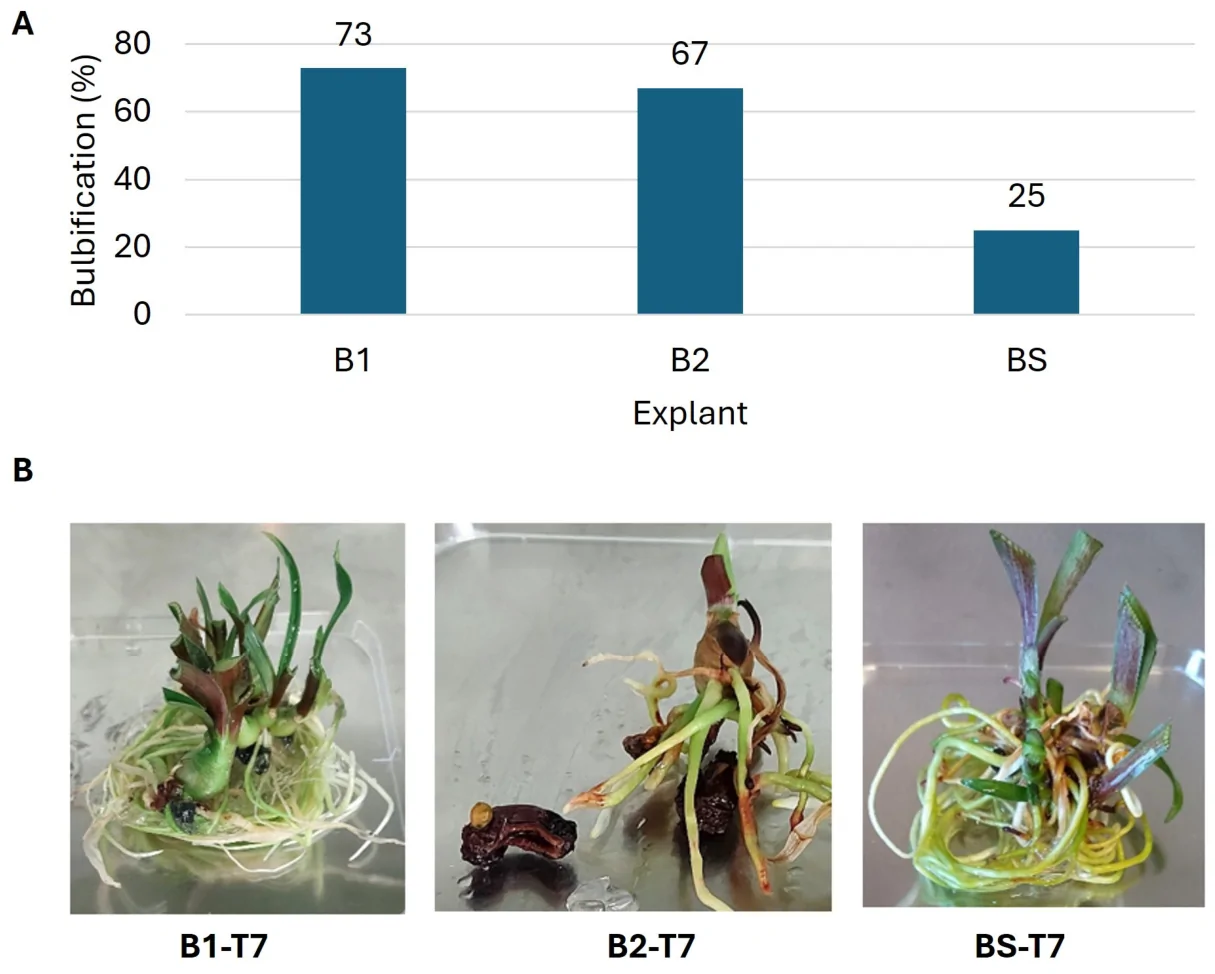
**Micropropagation and bulbification of regenerated *Hippeastrum × hybridum* lines.** (**A**) Frequency of confirmed bulbification among advanced regenerative series derived from the basal bulb fraction (B1), apical bulb fraction (B2), and ovary (BS). Percentages were calculated relative to the number of advanced regenerative series evaluated for each explant type (B1, 8/11; B2, 4/6; BS, 2/8) and therefore do not represent regeneration frequencies from the initial explants. (**B**) Representative *in vitro* cultures showing advanced regeneration and bulbous structure formation during the micropropagation stage, including the pronounced response observed in selected T7-derived lines at week 46. B1, basal bulb fraction including the basal plate; B2, apical bulb fraction without the basal plate; BS, ovary.

**Table 1:** Callogenic and organogenic responses of different *Hippeastrum × hybridum* explant types during *in vitro* culture.

Explant	Code	Callogenesis, *n*/N (%)	Organogenesis, *n*/N (%)	Main Developmental Outcome
Leaf	H	122/160 (76.3)	1/160 (0.6)	High callogenic response; isolated adventitious rooting without complete regeneration
Ovary	BS	90/120 (75.0)	9/120 (7.5)	High callogenic response with organogenic and regenerative competence in selected lines
Basal bulb fraction	B1	33/60 (55.0)	10/60 (16.7)	Intermediate callogenesis and the highest overall organogenic response
Floral scape	T	34/99 (34.3)	0/99 (0.0)	Callogenic response without subsequent organogenesis
Apical bulb fraction	B2	26/80 (32.5)	7/80 (8.8)	Moderate callogenesis with organogenic and regenerative competence
Stamen filaments from open flowers	AA	0/110 (0.0)	0/110 (0.0)	No callogenic or organogenic response
Stamen filaments from closed flower buds	BB	0/120 (0.0)	0/120 (0.0)	No callogenic or organogenic response
Isolated anthers	P	0/118 (0.0)	0/118 (0.0)	No callogenic or organogenic response
Root	R	0/140 (0.0)	0/140 (0.0)	Maintenance of the original tissue without callogenic or organogenic response

Note: Values represent the number of initially inoculated explants showing a confirmed response relative to the number of explants included in the corresponding consolidated dataset (*n*/N). Callogenesis and organogenesis were evaluated independently. Organogenesis was defined as the development of at least one newly formed organized structure, including roots, shoots, leaves, or bulblets. Structures subsequently generated through fragmentation or multiplication of regenerated material were not considered independent of initial response events. These response frequencies describe the observations obtained within the present experimental series and should not be interpreted as statistically validated estimates across independent biological batches or genotypes.

### 2.2. Organogenic Response and Micropropagation of Regenerated Lines

Organogenic competence in *Hippeastrum × hybridum* followed a pattern clearly distinct from callogenesis and was mainly associated with bulb- and ovary-derived explants (Figure 2B; Table 1). B1 showed the highest overall organogenic frequency, with 16.7% (10/60) of the initial explants producing at least one organized structure, followed by B2 with 8.8% (7/80) and BS with 7.5% (9/120). In contrast, H showed only a single adventitious rooting event (0.6%, 1/160), whereas T, AA, BB, P, and R did not develop new organogenic structures during the evaluated period.

Within B1, the strongest regenerative response was observed in T7, in which 4 of 6 explants (66.7%) developed organogenic structures, while the hormone-free control T0 responded in 3 of 6 explants (50.0%). T1, T2, and T9 also generated regenerative lines. In B2, organized regeneration was mainly associated with T0, T1, T2, and T9, with T2 showing the most consistent response across the initial experimental replicates. BS displayed a more treatment-dependent regenerative pattern, with the most relevant responses occurring in T2, T3, and T7. Thus, treatments promoting abundant callus formation did not necessarily favor subsequent organized regeneration.

Regenerative lines derived from B1, B2, and BS maintained organized growth during successive subcultures and progressed to the micropropagation stage. This phase was characterized by continued root and leaf development, thickening of regenerative basal structures, and, in selected lines, differentiation of new bulbous units (Figure 4). Among the advanced series evaluated, confirmed bulbification was observed in 8 of 11 B1 lines (72.7%), 4 of 6 B2 lines (66.7%), and 2 of 8 BS lines (25.0%) (Table S3; Figure 4A).

B1 showed the most sustained multiplication, with bulbous structures confirmed in T0, T1, T2, T7, and T9. The most pronounced response occurred in T7, where multiple bulbiform units developed together with active foliar and root growth. In B2, bulbification was confirmed in T0, T1, T7, and T9, whereas T2 showed marked root and vegetative development without unequivocal bulb differentiation. In BS, bulbification was more restricted and was confirmed in T2 and T7; the latter showed the most developed response, with multiple bulbous units associated with abundant root and leaf growth. Representative advanced regenerative cultures are shown in Figure 4B.

Overall, these results show that high callogenic competence was not necessarily associated with subsequent organogenic competence. While H exhibited one of the highest callogenesis frequencies but almost no organogenesis, B1, B2, and selected BS lines retained the capacity for organized regeneration and continued multiplication. This distinction between callus proliferation and regenerative competence provided complementary criteria for the subsequent selection and establishment of cell suspension cultures.

### 2.3. Establishment and Selection of Cell Suspension Lines

Among the callus-producing explants identified during the initial screening (Table 1), B1- and BS-derived cultures showed particularly rapid and abundant callus proliferation during successive subcultures on solid medium. Callus from H, B2, and T were also transferred to liquid medium; however, the limited amount of available biomass and its slower accumulation restricted further expansion of these cultures. Consequently, subsequent development of the cell suspension platform focused on B1 and BS, which provided sufficient biomass for repeated subculture and progressive scale-up.

Cell suspensions were initiated from B1 and BS calli maintained under either T6 or T8, while the corresponding callus lines continued to be maintained on their original solid media. Although T8 performed particularly well for callus growth and maintenance on solid medium, transfer to liquid culture revealed a different response. Under suspension conditions, both B1 and BS consistently showed greater visually assessed proliferation in T6 than in T8 suspension conditions. T6 was therefore selected as the maintenance medium for both suspension lines. These observations indicated that the hormonal conditions favoring callus proliferation on solid medium did not necessarily coincide with those supporting sustained biomass production in liquid culture.

Both selected lines adapted successfully to suspension culture and were progressively expanded until they reached 400 mL cultures in 1 L Erlenmeyer flasks, providing sufficient biomass for subsequent transfer to 2 L bioreactors. During flask-scale expansion, BS appeared to proliferate slightly faster than B1, although differences in growth performance between the two lines became more evident after transfer to bioreactor conditions, as described in Section 2.4.

A distinctive phenotypic feature emerged in the B1 suspension from the first liquid-culture subcultures. B1 cells developed a pale pink coloration that was rarely observed in routinely maintained callus on solid medium. This pigmentation became markedly more intense when subculture was delayed and became more intense during the period following COR addition. In contrast, BS suspensions retained a predominantly pale coloration.

Overall, the rapid and abundant biomass production observed for B1 and BS on solid medium, together with their successful adaptation to liquid culture and sustained growth under T6, supported their selection as the two cell lines used for subsequent bioreactor scale-up.

### 2.4. Scale-Up and Biomass Accumulation of Selected Cell Lines in 2 L Bioreactors

**Figure 5:**
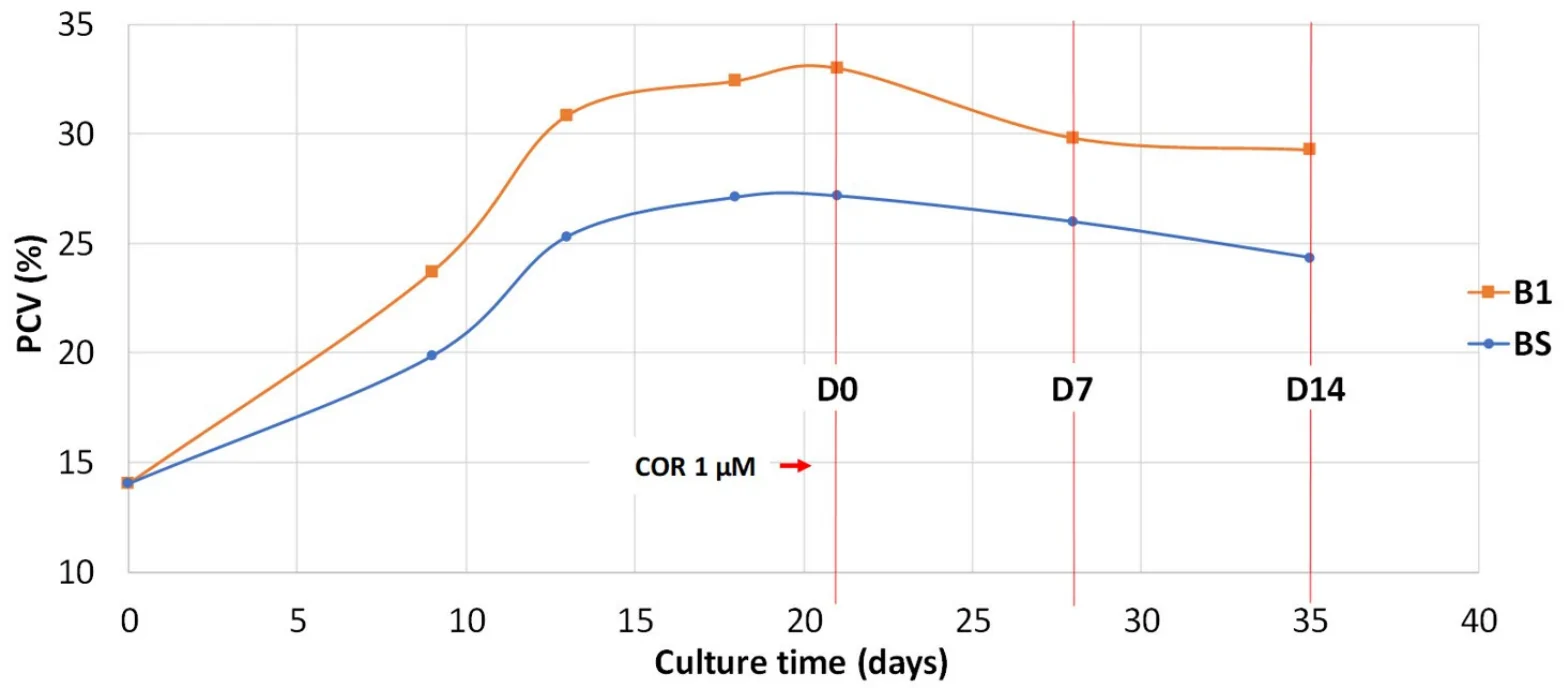
**Growth dynamics of B1 and BS *Hippeastrum × hybridum* cell suspension lines during cultivation and coronatine elicitation in 2 L bioreactors**. Packed cell volume (PCV) was monitored throughout the cultivation period. Both cultures were inoculated at an initial PCV of approximately 14% in a working volume of 1 L. After 21 days of growth, cultures were elicited with 1 µM coronatine (COR); this time point was designated D0. Vertical red lines indicate the sampling points immediately before COR addition (D0) and at 7 (D7) and 14 days (D14) after elicitation. Plotted PCV values correspond to the mean of three technical aliquots collected from the same culture at each sampling point. Each cell line was represented by a single bioreactor run; therefore, these values do not represent biological replication and were used for descriptive purposes only. B1, basal bulb-derived cell line; BS, ovary-derived cell line.

The selected B1 and BS cell suspensions were transferred to 2 L bioreactors at a working volume of 1 L and an initial packed cell volume (PCV) of approximately 14%. Both lines successfully adapted to bioreactor conditions and showed a progressive increase in PCV during the 21-day cultivation period (Figure 5).

In the bioreactor cultures evaluated, B1 showed greater estimated cell-biomass accumulation than BS. B1 PCV increased from 14.0 ± 0.11% at the beginning of bioreactor cultivation to 33.00 ± 0.45% after 21 days, the latter corresponding to D0 of the subsequent elicitation experiment. BS PCV increased from 14.0 ± 0.13% to 27.15 ± 0.20% over the same pre-elicitation cultivation period (Table 2). Based on the experimentally determined packed-cell densities of 1.097 g mL^−1^ for B1 and 1.091 g mL^−1^ for BS, these increases corresponded to estimated net biomass accumulations of approximately 208.4 g FW for B1 and 143.5 g FW for BS, equivalent to descriptive mean accumulation rates of 9.93 and 6.83 g FW day^−1^, respectively. These values represent estimates derived from the individual bioreactor run evaluated for each cell line and should not be interpreted as statistically validated growth parameters.

Biomass accumulation was accompanied by progressive changes in the physicochemical properties of the culture medium. Soluble solids decreased from 2.7 to 0.4 °Brix in B1 and from 2.6 to 0.6 °Brix in BS between the beginning of bioreactor cultivation and cultivation day 21. Electrical conductivity also declined, from 3586 to 2748 µS cm^−1^ in B1 and from 3610 to 3360 µS cm^−1^ in BS, with a more pronounced decrease in the B1 culture. The medium remained acidic throughout cultivation, reaching pH values of 3.79 in B1 and 3.64 in BS at D21.

Overall, both cell lines showed sustained growth and substantial biomass accumulation following scale-up to 2 L bioreactors. In the bioreactor runs evaluated, B1 displayed a greater increase in PCV and estimated net biomass accumulation than BS. The stabilization of the growth curves, together with the marked depletion of soluble solids, supported the selection of D21 as the transition point for coronatine elicitation.

### 2.5. Physiological Changes in Bioreactor-Grown Cell Suspensions During the COR-Treated Phase

After 21 days of bioreactor cultivation, when PCV accumulation had slowed and soluble solids were markedly depleted, the B1 and BS cell suspensions were exposed to 1 µM coronatine (COR). This time point was designated D0, and both lines were subsequently monitored for 14 days, with additional sampling at D7 and D14 (Figure 5; Table 3). During this period, both suspensions maintained relatively high PCV values, although a moderate decline was observed compared with D0. In B1, PCV decreased from 33.00 ± 0.45% at D0 to 29.27 ± 0.40% at D14, whereas in BS it declined from 27.15 ± 0.20% to 24.33 ± 0.18% over the same interval.

At D7, suspension was withdrawn to recover the cellular biomass and culture medium separately for alkaloid analysis. The removed volume was not replaced; therefore, the cultures were maintained at a lower working volume from D7 to D14 than before D7 sampling. Accordingly, measurements obtained after D7 were used primarily to describe the physiological evolution of the cultures rather than to estimate total bioreactor biomass production directly. Because working volume influences mixing, gas–liquid mass transfer, and the hydrodynamic environment of bioreactor cultures, D14 measurements should therefore be interpreted as describing the remaining culture under reduced-volume conditions rather than as a direct continuation of an equivalent constant-volume bioprocess.

Changes in PCV were accompanied by a further decrease in soluble solids and medium conductivity (Table 3). °Brix declined from 0.4 to 0.2 in B1 and from 0.6 to 0.3 in BS between D0 and D14. Conductivity showed a particularly marked decrease in B1, from 2748 to 2010 µS cm^−1^, whereas in BS it decreased more moderately, from 3360 to 3285 µS cm^−1^. These trends were consistent with continued physiological activity of both suspensions during the post-elicitation period.

In contrast, pH showed a clearly line-dependent pattern. In B1, pH progressively increased from 3.79 at D0 to 4.36 at D14, whereas in BS it initially decreased from 3.64 to 3.17 at D7, followed by a partial recovery to 3.28 at D14 (Table 3). These differences highlighted distinct physiological responses of the two cell lines during the period following elicitation.

**Figure 6:**
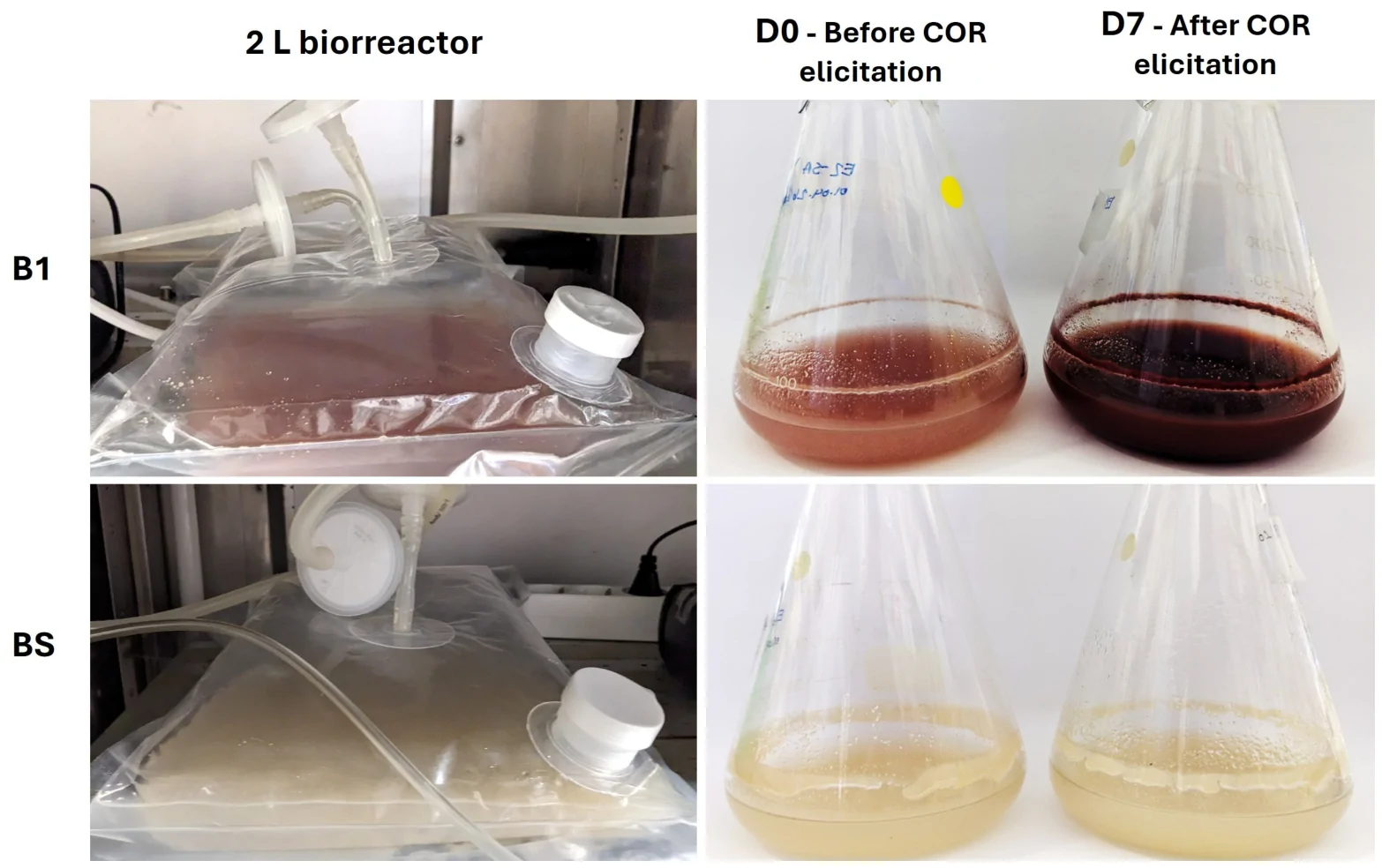
**Phenotypic characteristics of B1 and BS *Hippeastrum × hybridum* cell suspension cultures in 2 L bioreactors and following coronatine elicitation.** Representative appearance of B1 and BS cultures during bioreactor cultivation and immediately before (D0) and 7 days after (D7) elicitation with 1 µM coronatine (COR). B1 suspensions displayed a characteristic pale pink coloration at D0 that markedly intensified to a deeper reddish pigmentation by D7, whereas BS cultures remained predominantly pale and showed only slight browning. B1, basal bulb-derived cell line; BS, ovary-derived cell line.

During the period following COR addition, the two cell lines also displayed contrasting phenotypic changes (Figure 6). The B1 line, which already exhibited a characteristic pale pink coloration during suspension culture, showed a marked intensification of pigmentation and developed a deeper reddish appearance by D7. In contrast, BS remained predominantly pale, although slight browning was observed after elicitation.

Overall, both cell lines maintained a relatively high proportion of cellular biomass throughout the 14-day COR-treated period and displayed distinct temporal changes in physicochemical parameters and phenotype. The corresponding temporal changes in cellular and extracellular alkaloid-related profiles are presented in Section 2.6.1.

### 2.6. Alkaloid Profiling of Hippeastrum Cell Suspension Cultures

#### 2.6.1. Temporal Changes in the Alkaloid Extract Profiles of B1 and BS Bioreactor Cultures During the COR-Treated Phase

GC–MS profiling of cellular and extracellular alkaloid extracts revealed line-, fraction-, and time-dependent differences in the occurrence and relative abundance of structurally assigned compounds and unidentified signals. To characterize the temporal evolution of the alkaloid-related profiles during the COR-treated phase, cellular and extracellular fractions were analyzed independently at D0, D7, and D14. Structurally assigned compounds and their relative quantified values are summarized in Table 4, whereas the complete analytical dataset, including unidentified features, is provided in Tables S4 and S5. Values were expressed as µg Gal-eq mg^−1^ AE and were used for semi-quantitative comparative profiling only; they do not represent absolute compound-specific concentrations, biomass-normalized yields, volumetric yields, or alkaloid productivity. The structurally assigned compounds were not predefined analytical targets but corresponded to compounds that could be assigned from the GC–MS profiles obtained from the different sample types.

Before COR addition (D0), no quantifiable signals were detected in either the cellular or extracellular fractions of B1. In contrast, the BS culture medium already contained six unidentified signals, with a summed semi-quantitative signal of 16.516 µg Gal-eq·mg^−1^, whereas no signals were detected in the corresponding cellular fraction. Thus, the two selected lines displayed clearly different baseline profiles before elicitation.

The most pronounced changes were observed at D7. B1 developed a complex profile in both the culture medium and cellular biomass, reaching summed semi-quantitative signals of 58.350 and 51.169 µg Gal-eq·mg^−1^, respectively. Particularly relevant was the detection in the B1 extracellular fraction at D7 of hordenine (RT 11.50 min; RI 1469; 4.267 µg Gal-eq mg^−1^ AE) and *O*-methylnorbelladine (RT 24.66 min; RI 2602.3; 16.205 µg Gal-eq mg^−1^ AE). *O*-Methylnorbelladine is biosynthetically relevant because methylated norbelladine derivatives precede the oxidative phenol-coupling steps leading to major Amaryllidaceae alkaloid skeletons. However, the present assignment does not establish the position of O-methylation and should therefore not be reported specifically as 4′-*O*-methylnorbelladine without further confirmation. Hordenine was also structurally assigned in this fraction, but it is not interpreted here as a canonical Amaryllidaceae alkaloid skeleton or as a demonstrated intermediate of the Amaryllidaceae alkaloid biosynthetic pathway. The remaining quantified signals in B1 could not be structurally assigned and accounted for 37.878 µg Gal-eq·mg^−1^ in the medium and the entire quantified signal detected in the cellular fraction.

BS also showed a marked modification of its profile at D7. The summed signal in the extracellular fraction increased from 16.516 µg Gal-eq·mg^−1^ at D0 to 52.733 µg Gal-eq·mg^−1^ AE. This change reflected variation in the overall GC–MS profile; because the contributing features were not structurally assigned, they were not interpreted in relation to a specific alkaloid biosynthetic pathway.

By D14, the two cell lines displayed contrasting temporal patterns. In BS, the summed extracellular signal decreased to 12.930 µg Gal-eq·mg^−1^ AE and the cellular fraction decreased from 10.277 to 7.400 µg Gal-eq mg^−1^ AE. The composition of the unidentified features also changed over time, but these features were retained only as part of the complete analytical profile and were not assigned biological functions.

In B1, by contrast, quantified signals remained comparatively high at D14, reaching 46.155 µg Gal-eq·mg^−1^ in the medium and 44.215 µg Gal-eq·mg^−1^ in the cellular fraction. These values represented decreases of approximately 21% and 14%, respectively, relative to D7, but were accompanied by a clear qualitative reorganization of the profile. Hordenine and *O*-methylnorbelladine, which had been detected in the extracellular fraction at D7, were no longer detected at D14, whereas new unidentified signals appeared. A similar pattern was observed in the cellular fraction, where the number and identity of signals changed despite the relatively limited decrease in their summed quantified value.

The complete distribution of unidentified GC–MS features is provided in Table S4 for analytical transparency. Because these features could not be structurally assigned, they were not interpreted as specific alkaloids or used to infer regulation of Amaryllidaceae alkaloid biosynthesis. Overall, BS showed a transient maximum in quantified signals at D7 followed by a pronounced decline at D14, whereas B1 changed from an undetectable profile at D0 to a more complex extracellular and cellular profile at D7 that remained comparatively abundant, although compositionally reorganized, at D14. Given the absence of biological replication and of a time-matched non-elicited control, these temporal observations are descriptive and do not demonstrate a COR-specific effect.

#### 2.6.2. Alkaloid Profiles of *In Vitro* and *Ex Vitro* Plant Tissues

GC–MS analysis revealed distinct alkaloid extract profiles among *Hippeastrum × hybridum* tissues maintained *in vitro* and those collected from *ex vitro* plants. Structurally assigned compounds are summarized in Table 4, whereas the complete profiles are provided in Table S5. All tissues contained quantifiable signals, although their number, structural assignment, and relative abundance varied according to organ type and culture condition. Values were expressed as µg galantamine equivalents per mg of alkaloid extract (µg Gal-eq·mg^−1^) and therefore represent relative estimates rather than absolute concentrations of individual compounds.

The highest summed semi-quantitative signal was observed in in vitro leaves, reaching 17.975 µg Gal-eq·mg^−1^, followed by *ex vitro* bulbs at 16.964 µg Gal-eq·mg^−1^. Intermediate values were detected in *ex vitro* leaves (12.182 µg Gal-eq·mg^−1^) and *in vitro* bulbs (11.022 µg Gal-eq·mg^−1^), whereas *in vitro* roots showed the lowest summed value, at 6.386 µg Gal-eq·mg^−1^. These differences were accompanied by variations in profile complexity, with *in vitro* leaves and *ex vitro* bulbs displaying the largest number of quantifiable features.

Most detected signals could not be structurally assigned. Among the structurally assigned compounds, candimine, a homolycorine-type Amaryllidaceae alkaloid, was detected at RT 33.08–33.09 min (RIexp 3127.8) in all three *in vitro* tissues, reaching 2.223, 2.389, and 2.790 µg Gal-eq mg^−1^ AE in root, bulb, and leaf extracts, respectively. Candimine was also detected in *ex vitro* leaves at 2.685 µg Gal-eq mg^−1^ AE, whereas it was not detected in *ex vitro* bulbs. Thus, candimine showed a broad distribution among the *in vitro* tissues analyzed but a more restricted occurrence in the differentiated *ex vitro* material. Importantly, candimine was detected in this independent tissue-comparison dataset and not in the D0–D7–D14 bioreactor series; therefore, it was not evaluated as part of the temporal COR-treated experiment.

Unidentified features and their distribution among tissues are reported in Table S5 for completeness but were not assigned biological functions or interpreted as specific Amaryllidaceae alkaloids. Overall, comparison of *in vitro* and *ex vitro* tissues revealed differences in both the summed relative abundance and qualitative composition of the detected profiles, while the persistence of selected shared features, together with the occurrence of candimine in most tissues, indicates partial conservation of the metabolic profile across tissue types and culture conditions.

## 3. Discussion

### 3.1. Explant Origin Determines Callogenic and Regenerative Competence in Hippeastrum × hybridum

The *in vitro* response of *Hippeastrum × hybridum* was strongly influenced by explant origin, but callogenic and regenerative competence were not necessarily associated. H and BS showed the highest frequencies of callus formation, whereas B1, despite its lower overall callogenic response, displayed the highest organogenic competence. This contrast was particularly evident in H, where extensive callus formation was accompanied by almost no organized regeneration. Thus, callus proliferation alone was not a reliable predictor of subsequent morphogenic capacity.

Explant-dependent regeneration has previously been reported in *Hippeastrum*. Seabrook and Cumming [12] described regenerative responses from vegetative and floral tissues, whereas de Almeida et al. [13] demonstrated that floral tepals can retain substantial morphogenic competence under appropriate plant growth regulator combinations. Our results further show that explants exposed to the same range of hormonal treatments can follow markedly different developmental trajectories, reinforcing anatomical origin as a major determinant of *in vitro* response.

Bulb-derived tissues were particularly relevant because both B1 and B2 retained regenerative capacity, although B1 showed a clearly greater organogenic response. The presence of the basal plate in B1 may contribute to this behavior, as it represents a specialized region with considerable developmental activity. Recent studies in *H. papilio* have also demonstrated pronounced physiological and metabolic heterogeneity among bulb regions, including the basal plate [10,11]. Although these studies did not address morphogenesis directly, they reinforce the concept that the bulb is physiologically heterogeneous and that its different regions may respond differently under *in vitro* conditions.

Plant growth regulator composition further modulated these responses in an explant-dependent manner. Treatments promoting callus proliferation were not necessarily those favoring organized regeneration, as also reported in other Amaryllidaceae [15]. Conversely, the lack of sustained callogenic or organogenic response in AA, BB, P, and R should not be interpreted as an absolute lack of competence, but rather as the absence of an effective response under the conditions evaluated. Together, these results indicate that explant selection for a cell-based platform should consider not only initial callogenesis but also callus persistence, proliferation, morphology, and regenerative behavior.

### 3.2. Cell Line Selection and Transition from Solid Callus to Suspension Culture

The transition from callus to suspension culture represented a second and more restrictive selection step. Although several explant types produced callus, B1 and BS were particularly suitable because they combined sustained proliferation, sufficient biomass availability, and morphological characteristics compatible with transfer to liquid medium. H-, B2-, and T-derived calli were also tested in suspension, but their lower availability of proliferative biomass and slower accumulation limited further expansion. Thus, selection of B1 and BS reflected their performance throughout successive culture stages rather than their initial callogenesis frequencies alone.

An important finding was that the conditions favoring callus growth on solid medium were not necessarily optimal after transfer to liquid culture. T8 supported strong callus proliferation on solid medium, whereas both B1 and BS performed better in T6 under suspension conditions. Changes in nutrient and plant growth regulator availability, gas exchange, aggregation, and continuous contact with the medium can substantially alter cellular requirements after transfer to liquid culture. Similar line- and culture-format-dependent responses have been reported in *Narcissus* and other medicinal plant suspension systems [16,28]. These observations support a stepwise optimization strategy rather than the use of a single hormonal formulation throughout the entire culture process.

B1 also developed a reproducible pale-pink phenotype after transfer to liquid culture, which intensified when subculture was delayed and later during the elicitation phase. Anthocyanin biosynthesis and its transcriptional regulation have been described in *Hippeastrum × hybridum* flowers [29], suggesting that pigment-related specialized metabolism may be involved. However, because the pigment was not chemically characterized in the present cultures, its identity cannot be established. The coloration should therefore be interpreted as a line-specific reddish phenotype associated with adaptation to suspension culture rather than as evidence of anthocyanin accumulation.

Overall, the establishment of B1 and BS demonstrates that high callogenic competence, sustained callus proliferation, adaptation to liquid culture, and rapid biomass accumulation are related but distinct traits. The capacity to maintain growth across these transitions ultimately determined which lines were suitable for subsequent scale-up.

### 3.3. Scale-Up to 2 L Bioreactors Reveals Distinct Growth Behavior of B1 and BS Cell Suspension Cultures

Both B1 and BS were successfully transferred from shake-flask cultures to 2 L bioreactors and sustained biomass accumulation over the 21-day cultivation period. This represents a critical step because plant cell behavior at flask scale cannot necessarily be extrapolated to bioreactors, where mixing, aeration, oxygen transfer, and hydrodynamic conditions introduce additional constraints [21,22]. Adaptation to bioreactor conditions therefore constitutes an additional selection criterion in the development of plant cell biofactories.

Despite similar initial PCV values, B1 reached a higher final PCV and greater estimated net biomass accumulation than BS. This result is particularly relevant because BS had shown slightly faster proliferation during early flask culture, indicating that small-scale growth behavior did not fully predict performance after scale-up. Such line-dependent responses are common in plant cell systems and can arise from differences in aggregation, oxygen demand, nutrient uptake, or sensitivity to hydrodynamic conditions [21,23]. Under the conditions and individual bioreactor runs evaluated here, B1 showed greater estimated biomass accumulation than BS in the individual bioreactor runs evaluated. However, because each cell line was represented by a single bioreactor run, this difference should be interpreted descriptively and not as a statistically validated line-specific growth advantage.

Changes in soluble solids, conductivity, and pH provided complementary information on culture development. The decrease in soluble solids and conductivity accompanied biomass accumulation in both lines and was more pronounced in B1, consistent with its greater growth. However, pH followed distinct trajectories in B1 and BS, further supporting line-specific physiological behavior. These parameters cannot be assigned to individual metabolic processes, but their combined monitoring with PCV offers a practical approach for following the physiological state of the cultures.

By day 21, biomass accumulation had slowed while soluble solids had become markedly depleted, providing a practical point for transition from biomass production to metabolic elicitation. The cultures were therefore intentionally elicited at this stage, following the logic of a two-stage growth/production strategy in which biomass accumulation is first favored and specialized metabolism is subsequently stimulated. Such strategies are commonly applied in plant cell biotechnology because conditions supporting maximal growth do not necessarily coincide with those favoring specialized metabolite accumulation [1,25]. The successful establishment of both lines at the 2 L scale therefore provided a controlled platform for the subsequent evaluation of their metabolic response during the COR-treated phase [5].

### 3.4. Physiological and Phenotypic Responses During Coronatine Elicitation Are Line Dependent

Following 21 days of bioreactor cultivation, B1 and BS were exposed to 1 µM COR. Both retained relatively high PCV values during the subsequent 14 days, although moderate decreases were observed. These changes occurred together with continued variation in soluble solids, conductivity, and extracellular pH, indicating that the cultures remained physiologically dynamic during the elicitation phase. Importantly, the response differed between lines.

COR is widely used to stimulate specialized metabolism in plant cell cultures and can simultaneously affect growth and broader metabolic networks. In *Taxus*, COR activates taxane biosynthesis while also inducing wider changes in phenylpropanoid, terpenoid, and nitrogen-containing metabolism [25,27,30]. The moderate PCV decline observed in B1 and BS is therefore compatible with the physiological adjustments commonly associated with elicitation, although growth and metabolic responses cannot be expected to occur identically in different cell lines.

The contrasting pH responses of B1 and BS further emphasize this line dependence. B1 showed a progressive increase in extracellular pH, whereas BS initially became more acidic before partially recovering. Because pH integrates several processes, including ion uptake, proton exchange, nutrient assimilation, and metabolite release, these trajectories cannot be assigned to a single mechanism. Interpretation of the D14 response must additionally consider that approximately half of the culture volume was removed at D7 and was not replaced. Working volume is an important bioreactor operating parameter because changes in liquid volume can modify mixing, gas–liquid interfacial area, oxygen transfer, and the hydrodynamic environment experienced by the cells [21,31]. Although the remaining volume was within ranges commonly used in litre-scale bag-based bioreactor systems, the hydrodynamic conditions after D7 cannot be assumed to be equivalent to those at the initial 1 L working volume. Consequently, D14 measurements are best interpreted as describing the physiological and metabolic state of the remaining culture under reduced-volume conditions rather than as a continuous constant-volume bioprocess.

The clearest phenotypic response was observed in B1, where the pale-pink coloration became considerably more intense during the elicitation phase, whereas BS remained predominantly pale with limited browning. Although this response is compatible with activation of a stress-responsive pigment pathway, the pigment was not chemically identified and therefore cannot be attributed specifically to anthocyanins. Overall, the physiological and phenotypic changes observed during the COR-treated phase differed markedly between B1 and BS. COR is a well-established elicitor of specialized metabolism in plant cell cultures and can simultaneously affect growth and broader metabolic networks. In *Taxus* cell suspensions, 1 µM COR markedly enhanced taxane biosynthesis, increased the expression of pathway-related genes, and altered the distribution of metabolites between cellular and extracellular compartments. COR treatment has also been associated with broader metabolic reprogramming involving phenylpropanoid, terpenoid, and nitrogen-containing metabolites, while reductions or alterations in biomass accumulation have been reported in elicited cultures [25,27,30]. These observations indicate that changes in growth and physiology can accompany the activation of specialized metabolism during COR treatment. However, because the present experiment did not include a time-matched non-elicited bioreactor, the relative contribution of COR cannot be separated from culture-age-associated processes such as nutrient depletion and progression toward stationary-phase physiology.

### 3.5. Alkaloid Profiles and Biosynthetic Potential of Hippeastrum × hybridum Cell Cultures

The alkaloid extract profiles further demonstrated that specialized metabolism was strongly dependent on tissue identity, culture condition, and cell line. In vitro and *ex vitro* tissues showed both shared and tissue-specific signals, including a homolycorine-type signal detected in roots, bulbs, and leaves cultured *in vitro*, as well as in *ex vitro* leaves. This variability agrees with the pronounced spatial organization of Amaryllidaceae alkaloid metabolism described in *H. papilio*, where alkaloids and biosynthetic intermediates differ among leaves, roots, bulb regions, and specialized tissues [10,11]. Thus, establishment under *in vitro* conditions appeared to reorganize rather than simply suppress the metabolic profile.

Comparable metabolic plasticity has been reported in other Amaryllidaceae. Wild bulbs, *in vitro* bulbs, and callus cultures of *Rhodophiala pratensis* differed in the number and relative proportion of detected alkaloids, while plant growth regulator combinations influenced alkaloid composition in several related species [19,32]. Within *Hippeastrum*, micropropagation has also been associated with changes in lycorine biosynthesis [33]. These studies support the idea that differentiation state and culture conditions can substantially reshape specialized metabolism.

A further reorganization occurred after transition to dedifferentiated cell cultures. B1 and BS did not reproduce the profiles of their corresponding differentiated tissues and also differed markedly from each other. Similar reductions or changes in alkaloid diversity have been reported in Amaryllidaceae callus systems, including *Leucojum aestivum*, where dedifferentiated tissues retained biosynthetic competence despite a more restricted chemical profile [18]. Consequently, phytochemical performance should be considered alongside biomass accumulation when selecting cell lines for biofactory development.

The temporal behavior of B1 and BS during the elicitation phase reinforced this distinction. BS already contained extracellular signals at D0, reached its highest summed semi-quantitative signal at D7, and subsequently declined at D14. In contrast, B1 showed no quantifiable signals at D0 but developed a complex cellular and extracellular profile at D7 that remained comparatively abundant at D14, although its composition changed. This indicates that the two lines differed not only quantitatively but also in the temporal organization of their metabolism. Such dynamic metabolic rearrangements during COR-treated culture phases have also been described in *Taxus* cell cultures [26,27], although the absence of a time-matched control in the present study prevents direct attribution of these changes to COR.

Particularly relevant was the extracellular detection of *O*-methylnorbelladine in B1 at D7. Methylated norbelladine derivatives occupy a central position in Amaryllidaceae alkaloid biosynthesis, and 4′-*O*-methylnorbelladine is a key intermediate preceding the phenolic coupling reactions that generate several major alkaloid skeletons [20,34,35]. Detection of *O*-methylnorbelladine in the dedifferentiated B1 suspension is consistent with retention of metabolic competence related to the norbelladine branch of Amaryllidaceae alkaloid biosynthesis. However, the analytical assignment does not conclusively establish the methylation position, and the compound should not be identified specifically as the 4′-*O* isomer without further confirmation.

The extracellular occurrence of *O*-methylnorbelladine and hordenine may also be relevant from a bioprocess perspective, as recovery from the culture medium could potentially facilitate downstream extraction. Nevertheless, the present data cannot distinguish active secretion from transport, altered membrane permeability, or passive release from damaged cells. Hordenine is not interpreted here as a canonical Amaryllidaceae alkaloid skeleton or as a demonstrated biosynthetic intermediate, whereas *O*-methylnorbelladine is directly relevant to the norbelladine-derived pathway. Candimine, in contrast, is a homolycorine-type Amaryllidaceae alkaloid detected in the separate analysis of differentiated plant tissues and was not detected in the bioreactor time series. Thus, these compounds represent structurally assigned features from different sample sets rather than three predefined target metabolites, and no direct metabolic correlation among them can be inferred from the present dataset. Alkaloid transport and compartmentalization in Amaryllidaceae remain incompletely resolved, although recent studies support complex distribution across cellular, vascular, and extracellular compartments [10,11,35,36].

The phytochemical characteristics of the starting material should also be considered when evaluating the platform. *Hippeastrum × hybridum* represents horticultural material developed primarily for ornamental traits, and breeding within the genus has largely focused on characteristics such as flower size, shape, and colour rather than specialized metabolite composition [37]. Moreover, substantial variation in alkaloid composition has been demonstrated among ornamental *Hippeastrum* cultivars. The limited number of unequivocally assigned alkaloid-related signals observed here should therefore not be extrapolated to the metabolic potential of the genus as a whole [7].

A further limitation is that the entire experimental platform was derived from a single donor plant. Consequently, the explant-dependent developmental responses and chemical profiles observed here describe tissues and derived cell lines originating from one horticultural genotype and should not be extrapolated to *H. × hybridum* as a whole. Independent donor plants and additional genotypes will be required to determine the reproducibility of the platform and the extent of donor- or genotype-dependent variation.

This genotypic variability also provides a clear route for future improvement. Previous phytochemical surveys of *Hippeastrum* have revealed pronounced interspecific and intraspecific variation in alkaloid composition, including genotypes enriched in galantamine-type, lycorine-type, and homolycorine-type alkaloids [8,9]. In *H. papilio*, phytochemical selection has identified individuals with particularly favorable galantamine profiles, supporting its potential as an alternative industrial source [38]. The platform established here could therefore be transferred to species or genotypes selected beforehand for a target alkaloid or alkaloid profile. Combining phytochemical selection of the starting genotype with subsequent selection of high-performing cell lines may be considerably more effective than optimizing cultures derived from material originally selected for unrelated ornamental traits.

Overall, B1 and BS retained detectable metabolic competence throughout the transition from explant-derived material to suspension culture and bioreactor cultivation, but developed clearly line-specific chemical profiles. Among the individual bioreactor cultures evaluated, B1 combined greater estimated biomass accumulation with a more complex alkaloid-related profile during the COR-treated phase, including extracellular detection of hordenine and *O*-methylnorbelladine at D7. The present chemical data should therefore be interpreted as comparative and semi-quantitative and do not permit calculation of absolute alkaloid yield or productivity. Galantamine-equivalent values do not provide compound-specific concentrations, biomass-normalized yields, or volumetric productivities because individual analytes may differ in their GC–MS response. Most detected signals remain structurally unidentified. Unidentified GC–MS features were retained for analytical completeness but were not used to infer specific alkaloid biosynthetic responses. Therefore, the current results primarily demonstrate retained metabolic competence, temporal chemical reorganization, and differences between cellular and extracellular fractions. Accordingly, the present study should be regarded as a proof-of-concept characterization of the *Hippeastrum × hybridum* cell-culture platform rather than as a quantitatively validated production process. Additional structural confirmation, authentic standards, independent bioreactor replication, time-matched non-elicited controls, and quantitative process optimization will be required before reproducible production performance can be established.

## 4. Materials and Methods

### 4.1. Plant Material and General Culture Conditions

An adult ornamental plant of *Hippeastrum × hybridum* at the flowering stage, approximately 45 cm in height and bearing a bulb approximately 7 cm in diameter, was used as the initial plant material (Figure 1). The plant was purchased during the winter of 2024–2025 from Arte Japonés florist (Barcelona, Spain) and was sold with the European Union plant passport DE-NW-2100179, issued by Landgard Blumen & Pflanzen GmbH (Straelen-Herongen, Germany), with the internal traceability code CL-4601. The plant material was maintained under laboratory conditions until experimental processing. All explants and all callus, suspension, and bioreactor cultures generated in this study were derived from this single donor plant; consequently, donor-to-donor and genotype-level variation was not evaluated.

In vitro cultures were maintained on Murashige and Skoog (MS) basal medium containing vitamins, supplemented with 30 g L^−1^ sucrose and solidified with 2.7 g L^−1^ Gelrite. The medium was supplemented with 10 mL L^−1^ of an antioxidant solution containing 14.6 g L^−1^ L-glutamine, 2.5 g L^−1^ ascorbic acid, and 2.5 g L^−1^ citric acid. The pH was adjusted to 5.8 before autoclaving at 121 °C for 20 min. Unless otherwise specified for a particular experimental stage, cultures were maintained at 25 °C under a 16 h light/8 h dark photoperiod.

### 4.2. Surface Disinfection and Aseptic Establishment

#### 4.2.1. Preparation of Plant Material and Explant Collection

Before surface disinfection, the plant material was washed under running tap water for approximately 10 min to remove substrate residues and surface contaminants. In the case of the bulb, residues adhering to the outer scales were carefully removed using a soft brush under running water.

The plant was subsequently dissected to obtain the different explant types used for *in vitro* establishment: leaf (H), floral scape (T), ovary (BS), root (R), isolated anthers (P), and stamen filaments obtained from open flowers (AA) or closed flower buds (BB). The bulb was longitudinally sectioned and divided into a basal fraction containing the basal plate (B1) and an apical fraction without the basal plate (B2) (Figure 1). This nomenclature was maintained throughout the study to ensure traceability of the initial plant material and the derived lines.

#### 4.2.2. Surface Disinfection of Non-Bulbous Explants

Non-bulbous explants were immersed in 70% (*v*/*v*) ethanol for 30 s and subsequently rinsed three times with sterile distilled water. They were then incubated for 10 min in a 10 mg L^−1^ triadimenol solution prepared from a commercial stock solution, followed by three washes with sterile distilled water.

The explants were subsequently treated for 10 min under gentle agitation with a sodium hypochlorite solution prepared by diluting commercial bleach, containing approximately 3.7% NaOCl, to 30% (*v*/*v*), corresponding to a final NaOCl concentration of approximately 1.11%. The solution was supplemented with 0.01% (*v*/*v*) Tween 20. After disinfection, the explants were rinsed five times with sterile distilled water before *in vitro* establishment.

#### 4.2.3. Disinfection of Bulb Material

Because of the structural characteristics of bulb tissue, this material was subjected to a more intensive disinfection protocol performed over two consecutive days.

On the first day, the material was immersed in 70% (*v*/*v*) ethanol for 60 s and rinsed three times with sterile distilled water. It was then incubated for 10 min in a 10 mg L^−1^ triadimenol solution prepared from a commercial stock solution. During this treatment, vacuum was applied for 5 min using a commercial vacuum pump to facilitate penetration of the solution into the bulb tissue. The material was then rinsed three times with sterile distilled water and treated for 10 min with the NaOCl solution described above, corresponding to a 30% (*v*/*v*) dilution of commercial bleach (approximately 1.11% final NaOCl), supplemented with 0.01% (*v*/*v*) Tween 20. Finally, five consecutive washes with sterile distilled water were performed. After completion of the first-day procedure, the material was stored overnight in a sterile container in darkness until the second disinfection stage.

On the second day, the bulb material was again treated with 70% (*v*/*v*) ethanol for 60 s and rinsed three times with sterile distilled water. It was subsequently incubated for 10 min in a 1% (*w*/*v*) mercury (II) chloride (HgCl_2_) solution. After three washes with sterile distilled water, the material was subjected to a second 10 min treatment with the NaOCl solution (approximately 1.11% final concentration) supplemented with 0.01% (*v*/*v*) Tween 20. The procedure was completed with five consecutive washes with sterile distilled water before the establishment of the explants on culture medium.

### 4.3. Callus Induction and Plant Growth Regulator Treatments

The callogenic competence of the different *Hippeastrum × hybridum* explant types was evaluated using contrasting combinations of auxins and cytokinins. 2,4-Dichlorophenoxyacetic acid (2,4-D) and picloram were used as auxins, whereas 6-benzyladenine (BA) and thidiazuron (TDZ) were used as cytokinins.

Explants were established on the solid MS medium described in Section 4.1, supplemented with 30 g L^−1^ sucrose, 2.7 g L^−1^ Gelrite, and 10 mL L^−1^ of the antioxidant solution. Plant growth regulators were added to the medium before autoclaving according to the treatments listed in Table 5. The pH was adjusted to 5.8 before sterilization at 121 °C for 20 min. During the induction phase, cultures were maintained at 25 °C in darkness.

The screening included a control treatment without plant growth regulators (T0) and nine hormonal treatments (T1–T9), representing different auxin–cytokinin combinations and concentrations (Table 5). The distribution of these treatments among explant types depended on the availability of plant material and is described in detail in Section 4.4.1.

Explants and derived callus tissues were subcultured every 3 weeks onto fresh medium of the same hormonal composition as the original treatment. Callus formation was considered confirmed only when newly proliferating tissue, clearly distinguishable from the original explant and morphologically compatible with callus growth, was observed. Tissue swelling associated with the cut surface, wound healing, hydration, oxidation, or persistence of the original tissue was not considered callogenesis.

When callus masses were subsequently fragmented or transferred during subculture, the derived material retained the identity of the original explant and treatment. Therefore, callus masses generated through fragmentation or successive subcultures were considered part of the same experimental line and not as independent callus induction events.

### 4.4. Experimental Design and Evaluation of the In Vitro Response

The *in vitro* screening was designed to evaluate the response of different *Hippeastrum × hybridum* explant types to the plant growth regulator treatments described in Table 5. The evaluation included variables related to explant establishment and viability, callus induction and characteristics, organogenic response, and plant regeneration.

Traceability of each experimental unit was maintained from the initial establishment throughout subsequent subcultures. Fragments derived from the same explant or callus mass retained the identity of the original line and were not considered independent biological units.

#### 4.4.1. Experimental Design

The explants used during the establishment and induction phases included H, BS, AA, BB, P, T, B1, B2, and R (Figure 1).

The screening included the control treatment without plant growth regulators (T0) and the hormonal treatments described in Table 5. The range of treatments applied to each explant type depended on the availability of plant material. Whenever sufficient material was available, two initial culture plates were established for each explant–treatment combination. Plates were considered the primary experimental units, whereas individual explants represented observational units within each plate.

The initial number of explants was maintained as the reference throughout the experimental follow-up. Differences in the number of explants among particular treatments resulted from the availability and dimensions of the initial plant material. Response frequencies were calculated using the number of initially inoculated explants retained in the consolidated evaluable dataset for each explant–treatment combination. The nominal experimental design and the number of explants included in the final response analysis were recorded separately (Table 6). The complete treatment-specific response dataset is provided in Table S6.

#### 4.4.2. Response Variables During Establishment and Induction

The response of the explants was evaluated longitudinally throughout the establishment and induction phases and during subsequent subcultures. For each explant–treatment combination, survival, contamination, callogenesis, root formation, shoot formation, leaf development, bulblet formation, overall organogenic response, and, when applicable, complete plant regeneration were recorded.

Survival was defined as the persistence of visually viable tissue during the evaluation period. Contamination was recorded when visible microbial growth was confirmed on the plant material or culture medium.

Callogenesis was considered positive according to the criteria described in Section 4.3. Quantification was based on the initially inoculated explant; therefore, fragments subsequently generated through callus division or subculture were not considered additional induction events.

The frequency of each response was calculated according to the following general expression:

$$Response\;frequency\;\left(\%\right)=\frac{numer\;of\;explants\;showing\;the\;response}{numer\;of\;explants\;initially\;inoculated}\times 100$$

This calculation was applied independently to callogenesis, rooting, shoot formation, leaf development, bulblet formation, and other quantified responses.

The overall organogenic response was defined as the proportion of explants that developed at least one new organized structure, including roots, shoots, leaves, or bulblets. When a single explant developed more than one type of structure, each response was recorded separately for its corresponding variable, but the explant was counted only once when calculating the overall organogenic response.

Complete regeneration was evaluated independently from the overall organogenic response and was considered confirmed only when coordinated development of aerial and root structures compatible with a regenerated plant was observed. The exclusive formation of roots, leaves, incomplete shoots, or disorganized structures was classified as a partial morphogenic response.

When line traceability allowed the origin of regenerated material to be unequivocally established, regeneration was classified as direct when it originated from the initial explant without an identifiable intermediate callus phase, or indirect when regeneration occurred from previously induced callus tissue.

The temporal dynamics of the response were reconstructed from successive observations of each experimental line. Experimental week (W) was used as the main temporal reference. When a response was absent at one observation and present at the following evaluation, its appearance was assigned to the interval between both observations rather than retrospectively attributed to an exact day. For callogenesis, two stages were distinguished: the first detectable tissue proliferation compatible with a callogenic response and the first appearance of a clearly differentiated callus mass. This distinction allowed the onset of proliferative activity to be separated from the establishment of unequivocal callus tissue. The main quantitative, temporal, and qualitative response variables used throughout the *in vitro* evaluation are summarized in Table 7.

#### 4.4.3. Qualitative Characterization of Callus Tissue

Callus characteristics were evaluated independently from induction frequency by considering the degree of development, color, texture, friability, oxidation, and necrosis. These variables were assessed longitudinally for each experimental line while maintaining its identity according to the explant and treatment of origin.

Callus development was estimated using an ordinal scale from 0 to 5, where 0 indicated absence of callus, 1 incipient proliferation, 2 limited development, 3 moderate development, 4 abundant proliferation, and 5 extensive proliferation. This scale was used as a semi-quantitative assessment of visible tissue development and did not represent a gravimetric measurement of biomass.

Predominant callus color was classified as white, cream, yellow, orange, pink, red, green, brown, black, or mixed. Texture was described as compact, nodular, granular, semi-friable, friable, watery, mucilaginous, or heterogeneous.

Friability was additionally evaluated as a functional characteristic of the tissue and classified as confirmed, probable, non-friable, or undetermined. Friability was considered confirmed when the callus clearly disaggregated into smaller fragments or cell aggregates during manipulation or subculture.

Oxidation was evaluated using an ordinal scale from 0 to 5, where 0 indicated absence of oxidation and 5 represented generalized browning or extensive tissue blackening. Whenever possible, the predominant location of oxidation was also recorded, including the cut surface, explant margins, callus tissue, original explant tissue, or culture medium. Necrosis was recorded separately as a visual estimate of the proportion of apparently non-viable tissue.

Cell lines for subsequent establishment of suspension cultures were selected by jointly considering sustained growth during successive subcultures, degree of proliferation, morphological stability, friability, and low or absent tissue oxidation.

### 4.5. Micropropagation and Maintenance of Regenerated Material

Regenerated bulbous structures maintained *in vitro* were used as the starting material for the micropropagation stage. Bulbs with an approximate diameter of 1.5 cm were selected for the division assays. Before sectioning, developed leaves and roots were removed to facilitate handling and obtain comparable propagation units.

The bulbs were longitudinally divided into 4, 8, or 12 segments, ensuring that each segment retained a portion of the basal region. The resulting propagation units were established on the control treatment T0, corresponding to the MS medium described in Section 4.1 without plant growth regulators, and maintained under the general culture conditions described above.

Subcultures were performed according to the growth and development of each line, particularly when the plant material occupied the available space within the culture vessel or showed signs of medium depletion. In general, the lines were subcultured at approximately 1–2-month intervals, without exceeding 2 months between successive subcultures.

During the micropropagation stage, the development of roots, shoots, leaves, and new bulbous structures was recorded, together with the ability of the lines to maintain organized growth over successive subcultures. Traceability of the original explant, treatment, and experimental line was maintained throughout the process.

When regenerated structures were subsequently fragmented to continue multiplication, the resulting units were considered part of the same experimental line and not as independent regeneration events. Thus, the initial regenerative response was distinguished from the subsequent multiplication capacity of each line.

### 4.6. Establishment and Maintenance of Cell Suspension Cultures

Callus lines selected on the basis of sustained growth, morphological stability, and friability were used to establish cell suspension cultures. Fresh callus tissue was inoculated into liquid MS medium at a ratio of 1 g fresh weight per 10 mL of medium. During the initial establishment of the suspensions, each cell line was maintained in the same plant growth regulator treatment from which the corresponding callus line originated.

Cultures were maintained at 25 °C in darkness under continuous orbital agitation at 120 rpm. Cell suspensions were subcultured every 7 days. For medium renewal, cultures were allowed to sediment under sterile conditions and the spent medium was carefully removed by decantation, minimizing disturbance of the cell aggregates. Fresh liquid medium was then added to restore the initial working volume.

During the early stages of suspension establishment and expansion, when culture volume and biomass availability were still limited, growth was monitored by determining the sedimented cell volume. For this purpose, the entire culture was temporarily transferred to sterile graduated cylinders and allowed to sediment, after which the volume occupied by cells and cell aggregates was recorded relative to the total suspension volume. Following measurement, the cells were recovered and returned to the culture, allowing biomass monitoring without compromising the continuity of the suspension.

When the sedimented cell volume reached approximately 30% (*v*/*v*), the suspensions were progressively transferred to larger Erlenmeyer flasks. At each scale-up step, the initial cell volume was adjusted to approximately 10% (*v*/*v*) of the final culture volume by adding fresh medium, thereby maintaining an appropriate balance between biomass, nutrient availability, and aeration.

Progressive scale-up was continued until cultures reached 1 L Erlenmeyer flasks with a working volume of 400 mL. From this scale onwards, when sufficient biomass was available to permit sampling without compromising culture continuity, growth was monitored using packed cell volume (PCV), determined by centrifugation as described for the bioreactor scale-up stage in Section 4.7.

Cell lines showing stable growth and suitable culture characteristics during this phase were selected for subsequent transfer to 2 L bioreactors.

### 4.7. Scale-Up of Cell Suspension Cultures in 2 L Bioreactors

Selected cell suspension lines from the flask expansion stage were transferred to custom-designed 2 L disposable bag bioreactors developed in the Plant Physiology Laboratory. Prior to transfer, the selected lines had been established and maintained in 1 L Erlenmeyer flasks.

Each bioreactor was inoculated to an initial PCV of approximately 14% (*v*/*v*), with a final working volume of 1 L. Cultures were maintained at 25 °C in darkness, continuously aerated with ambient air at a flow rate of 2 L min^−1^, and agitated at 35–40 rpm.

Packed-cell density was determined gravimetrically using centrifuged 10 mL suspension aliquots. After centrifugation, the supernatant was removed and the fresh cell pellet was weighed. Packed-cell volume was estimated from the difference between the initial suspension volume and the recovered supernatant volume. Cell density was calculated as the fresh pellet mass divided by the estimated packed-cell volume (g mL^−1^).

Cell growth was monitored periodically by determining the PCV. At each sampling point, three 10 mL aliquots were collected from each culture and centrifuged at 5000 rpm (3333× *g*) for 15 min. After centrifugation, the supernatant was carefully removed and the volume occupied by the packed cell fraction was recorded. The three aliquots were considered technical measurements of the same culture and were used to estimate the mean PCV at each sampling time. Each cell line was represented by a single bioreactor culture; consequently, PCV measurements were used descriptively and no inferential comparison between cell lines was performed.

In addition to PCV, culture pH, soluble solids content (°Brix), and electrical conductivity were monitored throughout the cultivation period. These parameters, together with PCV evolution, were used to characterize cell growth and the physiological status of the cultures during scale-up and to determine the appropriate stage for the elicitation treatment described in Section 4.8.

### 4.8. Coronatine Elicitation

Once the cell cultures reached the stationary growth phase, as determined from PCV evolution and the physicochemical parameters described in Section 4.7, coronatine (COR) was aseptically added to a final concentration of 1 µM. Both selected cell lines were subjected to the same elicitation treatment.

The first sampling was performed immediately before COR addition and was defined as D0. Subsequent samplings were carried out at 7 days (D7) and 14 days (D14) after elicitation. Following COR addition, cultures were maintained under the same conditions described for bioreactor cultivation in Section 4.7.

At each sampling point, PCV was determined according to the procedure described in Section 4.7, and the culture pH, soluble solids content (°Brix), and electrical conductivity were recorded.

The elicitation experiment was followed longitudinally using the same bioreactor for each cell line. At D7, approximately 500 mL of cell suspension was withdrawn for recovery of biomass and culture medium samples, without subsequent replacement of the removed volume while agitation and aeration settings were maintained unchanged. Therefore, between D7 and D14, the cultures were maintained at a lower working volume than at the beginning of the elicitation period. At D14, the remaining material in each bioreactor was harvested.

At each sampling point, cell biomass and culture medium were recovered and processed as separate fractions for subsequent alkaloid extraction and analysis, allowing compounds associated with the cellular biomass to be distinguished from those present in the extracellular fraction.

### 4.9. Alkaloid Extraction and Sample Preparation

Alkaloids were extracted separately from cell biomass and culture medium to keep the cellular and extracellular fractions distinct throughout the analysis. In addition, leaf, root, and bulb samples from *Hippeastrum × hybridum* plants grown *in vitro* and *ex vitro* were processed for subsequent alkaloid characterization.

Cell suspension samples were centrifuged to separate the biomass from the culture medium. The recovered biomass was lyophilized, and the dried material was subjected to three consecutive methanol extractions using 50 mL of methanol per gram of lyophilized material. Each extraction was performed by sonication for 30 min. The methanolic fractions were combined and the solvent was removed under reduced pressure using a rotary evaporator. The resulting dry residue was resuspended in a 2% (*v*/*v*) aqueous H_2_SO_4_ solution using 20 mL of acid solution per gram of the initial lyophilized plant material.

Leaf, root, and bulb samples from plants grown *in vitro* and *ex vitro* were lyophilized and processed following the same procedure described for the cell biomass.

Culture medium fractions were directly acidified by adding concentrated H_2_SO_4_ (98%) to a final concentration of 2% (*v*/*v*).

The acidified aqueous fractions obtained from both the cell biomass and the culture medium were subjected to three consecutive washes with dichloromethane (DCM) using a 1:1 ratio between the acidified aqueous phase and DCM. After each wash, the phases were separated using a separatory funnel; the organic phase was discarded and the acidified aqueous phase was retained.

The aqueous phase was subsequently basified by adding 30% NH_4_OH until a pH of approximately 9.0 was reached. Alkaloids in their free-base form were then extracted by three consecutive extractions with DCM using a 1:2 DCM-to-basified aqueous phase volume ratio. After each extraction, the phases were separated using a separatory funnel, and the organic phases were recovered and combined. The DCM was evaporated to dryness, and the resulting alkaloid extracts were stored until GC–MS analysis, as described in Section 4.10.

### 4.10. GC–MS Analysis, Alkaloid Identification, and Relative Quantification

The alkaloid extracts obtained as described in Section 4.9 were redissolved in methanol to a final concentration of 2 mg mL^−1^. Codeine in methanol was used as the internal standard. Aliquots of alkaloid extract and internal-standard solution were combined according to the analytical series, maintaining the corresponding sample-to-internal-standard proportion used for quantitative processing. After homogenization, 1 µL of each prepared sample was injected into the GC–MS system. The preparation ratios and corresponding quantitative corrections are described in Section 4.10.3.

#### 4.10.1. GC–MS Analysis

Analyses were performed using an Agilent 6890N gas chromatograph coupled to an Agilent 5975 mass spectrometer and equipped with an Agilent 7683B autosampler, all from Agilent Technologies (Santa Clara, CA, USA). Separation was carried out on a TR-45232 Sapiens-X5MS capillary column (30 m × 0.25 mm × 0.25 µm), using helium as the carrier gas at a constant flow rate of 1.0 mL min^−1^. Injection was performed in splitless mode and electron impact ionization (EI) was carried out at 70 eV.

The oven temperature program started at 100 °C and was held for 12 min, followed by an increase to 180 °C at 15 °C min^−1^, with a 1 min hold. The temperature was then increased to 300 °C at 5 °C min^−1^ and held for 10 min. The injector temperature was set at 250 °C and the detector temperature at 280 °C [39].

#### 4.10.2. Compound Assignment and Reporting of Unidentified GC–MS Features

Chromatograms were processed and deconvoluted using the Automated Mass Spectral Deconvolution and Identification System (AMDIS, version 2.64; National Institute of Standards and Technology, NIST). Compound assignments followed the GC–MS methodology previously applied by our group to Amaryllidaceae alkaloid profiling [8,40,41]. Briefly, assignments were based on the combined evaluation of electron-impact (EI) mass-spectral fragmentation patterns and experimental retention indices (RI). Experimental data were compared primarily with the specialized Amaryllidaceae alkaloid reference library developed by the Natural Products and Plant Biotechnology Research Group at the University of Barcelona. The authentic reference compounds included in this library were previously isolated and structurally characterized using complementary spectroscopic techniques, including nuclear magnetic resonance (NMR), ultraviolet spectroscopy (UV), circular dichroism (CD), and mass spectrometry (MS). The NIST Mass Spectral Library and previously published spectral data were used as additional supporting sources.

Experimental RI values were determined relative to a C9-C30 n-alkane standard mixture analyzed under the same chromatographic conditions, following the previously established methodology [8]. Structural assignments were retained when the EI fragmentation pattern and available chromatographic information were consistent with the corresponding reference data. The compounds reported in the present study were not confirmed by direct co-injection of authentic standards within the same analytical sequence and should therefore be interpreted as library-supported structural assignments. Where the available analytical information did not allow discrimination between closely related positional or structural isomers, assignment was retained at a broader structural level. Accordingly, the signal assigned to *O*-methylnorbelladine was not attributed specifically to the 4′-*O*-methyl isomer.

Signals lacking sufficient spectral and chromatographic support for reliable structural assignment were retained as unidentified GC–MS features for comparative profiling and were not classified as alkaloids solely on the basis of their occurrence in alkaloid-enriched extracts. For these features, labels such as “m/z 107/55” indicate characteristic fragment ions observed in the deconvoluted EI mass spectrum that were used, together with retention time and RI, to distinguish and track individual features among samples. The slash separates the reported ions and does not represent an ion-abundance ratio.

This approach follows the GC–MS identification strategy previously applied by our group for Amaryllidaceae alkaloid profiling in *Clinanthus*, *Hippeastrum*, and *Narcissus* species [8,40,41].

#### 4.10.3. Semi-Quantitative Estimation Using Galantamine Equivalents

Semi-quantitative estimation was performed using a galantamine calibration curve prepared at concentrations of 10, 20, 40, 60, 80, and 100 µg mL^−1^, with codeine at 25 µg mL^−1^ as the internal standard. Peak areas obtained after GC–MS data processing were normalized to the internal-standard response and used to estimate the relative abundance of detected compounds and features by interpolation from the galantamine calibration relationship. This approach follows the comparative GC–MS methodology previously applied to Amaryllidaceae alkaloid profiling [8,40].

Samples prepared using a 1:1 extract-to-internal-standard ratio were processed directly using the corresponding calibration relationship. For the E2 analytical series, prepared using a 3:1 extract-to-internal-standard ratio, the difference in sample proportion was incorporated into the calculation after inversion of the calibration equation.

Results were expressed as micrograms of galantamine equivalents per milligram of alkaloid extract (µg Gal-eq mg^−1^ AE). Except for galantamine itself, these values represent semi-quantitative relative estimates based on the analytical response obtained with the galantamine calibration curve. No compound-specific calibration curves or analyte-specific response factors were established for the other detected compounds. Because individual analytes may differ in ionization efficiency, fragmentation behavior, and detector response, the reported Gal-eq values should not be interpreted as absolute compound-specific concentrations, total alkaloid contents, biomass-normalized yields, or volumetric productivities. They were used exclusively for comparative profiling of samples processed and analyzed under the same experimental conditions.

Cell biomass and culture medium fractions were analyzed independently. Temporal profiles at D0, D7, and D14 were compared descriptively on the basis of structurally assigned compounds, unidentified GC–MS features, and their semi-quantitative galantamine-equivalent responses.

### 4.11. Data Analysis

Data from *in vitro* establishment, callogenesis, and morphogenic responses were analyzed using absolute counts and response frequencies calculated relative to the actual number of explants initially inoculated for each explant–treatment combination. Initial culture plates were considered the primary experimental units, whereas individual explants were treated as observational units. Derived fragments, successive subcultures, and callus masses originating from the same line were used for longitudinal follow-up but were not considered independent biological replicates.

Morphological and qualitative variables were analyzed descriptively by considering the frequency, magnitude, persistence, and temporal evolution of the responses. Data from cell suspensions and bioreactor cultures were also evaluated descriptively. Aliquots used for PCV determination were considered technical measurements of the same culture rather than independent biological replicates. Accordingly, no inferential statistical tests were applied to comparisons between bioreactor cultures.

Changes recorded between D0, D7, and D14 following coronatine addition were interpreted as temporal variations within the same culture. In the absence of a parallel non-elicited control, these changes were not attributed exclusively to COR. Alkaloid profiles were compared descriptively among cell lines, sampling times, and cellular and extracellular fractions.

## 5. Conclusions

This study established an integrated *Hippeastrum × hybridum* cell-culture platform spanning explant selection, callus induction, suspension establishment, and an intermediate 2 L bioreactor scale-up stage. Explant origin strongly influenced callogenic and morphogenic competence, and progressive selection identified B1 and BS as the most suitable lines for suspension culture and scale-up. Both lines maintained growth following transfer to bioreactor conditions but displayed distinct growth, physiological, and metabolic behaviors, with B1 showing greater estimated net biomass accumulation in the bioreactor cultures evaluated.

Alkaloid profiling further revealed line- and time-dependent metabolic differences during the elicitation phase. Notably, B1 developed a complex cellular and extracellular alkaloid-related profile and hordenine and *O*-methylnorbelladine were detected in the extracellular fraction at D7, consistent with retention of metabolic competence related to Amaryllidaceae alkaloid biosynthesis. Although further structural confirmation, independent biological replication, and quantitative optimization are required, the present platform provides a proof-of-concept basis for future development of controlled *Hippeastrum* cell-culture systems. Its application to genotypes preselected for pharmaceutically relevant alkaloid profiles could combine phytochemical selection with cell-line optimization, scale-up, and elicitation.

## Figures and Tables

**Table 2:** Descriptive growth performance and estimated biomass accumulation of the B1 and BS cell suspension cultures evaluated after 21 days in 2 L bioreactors.

Cell Line	Initial PCV (%)	PCV at D21 (%)	Net PCV Increase (mL)	Estimated Net Biomass Accumulation (g fw)	Mean Biomass Accumulation Rate (g fw day^−1^)
BS	14.0 ± 0.13	27.15 ± 0.20	131.5	143.5	6.83
B1	14.0 ± 0.11	33.00 ± 0.45	190.0	208.4	9.93

Note: Net biomass accumulation was estimated from the increase in PCV between the beginning of bioreactor cultivation and cultivation day 21, using experimentally determined packed-cell densities of 1.091 g mL^−1^ for BS and 1.097 g mL^−1^ for B1. PCV values are expressed as mean ± SD of three technical aliquots obtained from the same bioreactor culture at each sampling point. Each cell line was evaluated in a single bioreactor run; therefore, biomass accumulation and daily accumulation rates are descriptive estimates and were not subjected to inferential statistical analysis.

**Table 3:** Physiological parameters of B1 and BS *Hippeastrum × hybridum* cell suspension cultures following coronatine elicitation.

Cell Line	Sampling Time	PCV (%)	pH	Soluble Solids (°Brix)	Conductivity (µS cm^−1^)
BS	D0	27.15 ± 0.20	3.64	0.6	3360
BS	D7	25.97 ± 0.30	3.17	0.4	3311
BS	D14	24.33 ± 0.18	3.28	0.3	3285
B1	D0	33.00 ± 0.45	3.79	0.4	2748
B1	D7	29.80 ± 0.25	4.29	0.3	2393
B1	D14	29.27 ± 0.40	4.36	0.2	2010

Note: D0 corresponds to the sampling point immediately before the addition of 1 µM coronatine (COR), after 21 days of bioreactor cultivation. D7 and D14 correspond to 7 and 14 days after elicitation, respectively. PCV values are expressed as mean ± SD of three technical aliquots obtained from the same culture. Approximately 500 mL of each cell suspension was harvested at D7 for separate recovery of cellular biomass and culture medium; the removed volume was not replaced.

**Table 4:** Structurally assigned compounds detected by GC–MS in *Hippeastrum × hybridum* cell cultures and plant tissues.

Sample	Sampling Point/Tissue	Fraction	Compound Assignment	RT (min)	RI	Gal-Equivalent Response (µg Gal-eq mg^−1^ AE)
B1	D7	Culture medium	Hordenine	11.5	1469	4.267
B1	D7	Culture medium	*O*-Methylnorbelladine	24.66	2602.3	16.205
Plant tissue	*in vitro* root	Cellular extract	Candimine	33.08	3127.8	2.223
Plant tissue	*in vitro* bulb	Cellular extract	Candimine	33.08	3127.8	2.389
Plant tissue	*in vitro* leaf	Cellular extract	Candimine	33.08	3127.8	2.79
Plant tissue	*ex vitro* leaf	Cellular extract	Candimine	33.08	3127.8	2.685

Note: Values are expressed as µg galantamine equivalents per mg of alkaloid extract (µg Gal-eq mg^−1^ AE) and represent relative analytical estimates rather than absolute compound-specific concentrations. RT, retention time; RI, experimental retention index; AE, alkaloid extract. *O*-Methylnorbelladine was assigned at the level of the methylated norbelladine structure; the position of *O*-methylation could not be conclusively established by the present analysis. Complete GC–MS profiles, including unidentified signals, are provided in Tables S4 and S5.

**Table 5:** Composition of plant growth regulator treatments used for callus induction in *Hippeastrum × hybridum*.

Treatment	Auxin	Auxin Concentration (mg L^−1^)	Cytokinin	Cytokinin Concentration (mg L^−1^)
T0	—	0	—	0
T1	2,4-D	0.5	BA	0.5
T2	2,4-D	1.0	BA	1.0
T3	2,4-D	2.0	BA	2.0
T4	Picloram	0.5	TDZ	0.5
T5	Picloram	1.0	TDZ	1.0
T6	Picloram	2.0	TDZ	2.0
T7	2,4-D	1.0	BA	0.5
T8	Picloram	1.0	TDZ	0.5
T9	2,4-D	0.5	BA	1.0

Note: 2,4-D, 2,4-dichlorophenoxyacetic acid; BA, 6-benzyladenine; TDZ, thidiazuron. T0 corresponded to the control medium without plant growth regulators.

**Table 6:** Treatment allocation and initial number of explants used in the *in vitro* screening of *Hippeastrum × hybridum*.

Explant	Code	Treatments	N per Treatment	No. of Treatments
Leaf	H	T0–T9	20	10
Ovary	BS	T0–T9	20	10
**STAMEN FILAMENTS FROM OPEN FLOWERS**	AA	T0–T6	20	7
Stamen filaments from closed flower buds	BB	T0–T6	20	7
Isolated anthers	P	T0–T6	20 ^1^	7
Floral scape	T	T0–T9	10 ^2^	10
Basal bulb fraction	B1	T0–T9	6	10
Apical bulb fraction	B2	T0–T9	8	10
Root	R	T0–T9	14	10

Note: Two initial culture plates were established for each explant–treatment combination whenever sufficient plant material was available. ^1^ For P, 20 explants were established per treatment, except for T6, which comprised 18 explants. ^2^ For T, T0 comprised 9 explants, whereas T1–T9 comprised 10 explants per treatment. T0 corresponded to the control treatment without plant growth regulators.

**Table 7:** Variables used to evaluate the *in vitro* response of *Hippeastrum × hybridum*.

Variable	Type of Assessment	Reference Unit	Main Criterion
Survival	Binary/proportional	Initial explant	Persistence of visually viable tissue
Contamination	Binary/proportional	Explant/plate	Confirmed visible contamination
Callogenesis	Binary/proportional	Initial explant	Formation of new callus tissue
Callus development	Ordinal	Callus line	Visual development, 0–5 scale
Color	Categorical	Callus line	Predominant tissue color
Texture	Categorical	Callus line	Morphological organization of the tissue
Friability	Categorical	Callus line	Capacity for tissue disaggregation
Oxidation	Ordinal	Explant/callus	Browning intensity, 0–5 scale
Necrosis	Semi-quantitative	Explant/callus	Visual proportion of apparently non-viable tissue
Organogenesis	Binary/proportional	Initial explant	Formation of roots, shoots, leaves, or bulblets
Complete regeneration	Binary	Explant/line	Coordinated development of aerial and root structures
Response time	Temporal	Experimental line	First week or interval in which the response was confirmed

## Data Availability

The data presented in this study are available in the article and Supplementary Materials. Additional data supporting the findings of this study are available from the corresponding author upon reasonable request.

## Supplementary Materials

The following supporting information can be downloaded at https://www.mdpi.com/article/10.3390/plants15193041/s1.

## Abbreviations

The following abbreviations are used in this manuscript:

AA	Stamen filament from open flower
AE	Alkaloid extract
AMDIS	Automated Mass Spectral Deconvolution and Identification System
B1	Basal bulb fraction including the basal plate
B2	Apical bulb fraction without the basal plate
BA	6-Benzyladenine
BB	Stamen filament from closed flower bud
BS	Ovary
COR	Coronatine
D0	Sampling time immediately before COR addition
D7	Seven days after COR elicitation
D14	Fourteen days after COR elicitation
DCM	Dichloromethane
EI	Electron ionization
FW	Fresh weight
Gal	Galantamine
Gal-eq	Galantamine equivalents
GC–MS	Gas chromatography–mass spectrometry
H	Leaf
MeOH	Methanol
MS	Murashige and Skoog medium
NIST	National Institute of Standards and Technology
P	Anther
PCV	Packed cell volume
PGR	Plant growth regulator
PIC	Picloram
R	Root
RI	Retention index
RT	Retention time
T	Floral scape
TDZ	Thidiazuron
2,4-D	2,4-Dichlorophenoxyacetic acid
