# Supplementary material for: From Explant Selection to Bioreactor Cultivation: Development of a Hippeastrum × hybridum Cell Culture Platform for Amaryllidaceae Alkaloid-Related Metabolism
Source: Plants (Basel). 2026 Oct 4;15(19):3041. doi: 10.3390/plants15193041 (PMC13667019; doi:10.3390/plants15193041)
Supplement: Supplementary file 1 [file plants-15-03041-s001.zip › plants-4554271-supplementary.pdf]

Supplementary Materials

From Explant Selection to Bioreactor Cultivation: Development of a *Hippeastrum* × *hybridum* Cell Culture Platform for Amaryllidaceae Alkaloid-Related Metabolism

María Lenny Rodríguez-Escobar [1], Laura Torras-Claveria [1] and Diego Hidalgo-Martínez [1,2,*]

[1] Department of Biology, Healthcare and the Environment, Faculty of Pharmacy and Food Sciences, University of Barcelona, 08028 Barcelona, Spain; m-rodriguez@ub.edu (M.L.R.-E.); lauratorrascl@ub.edu (L.T.-C.)
[2] Maria de Maeztu Unit of Excellence, Institute of Nutrition and Food Safety, University of Barcelona (INSA-UB), 08921 Santa Coloma de Gramenet, Spain
* Correspondence: dhidalgo@ub.edu

Table S1. Physiological behavior of *Hippeastrum × hybridum* explants during *in vitro* establishment.

EXPLANT	TREATMENTS EVALUATED	INITIAL TISSUE PRESERVATION	OXIDATION DURING ESTABLISHMENT	NECROSIS/DETERIORATION	OVERALL PHYSIOLOGICAL RESPONSE
**B1**	T0–T9	High in most lines	Initially low to moderate; progressive in specific lines	Localized in some combinations	Good initial preservation, with line-dependent deterioration during prolonged culture
**B2**	T0–T9	High	Generally low to moderate during the first evaluations	Initially limited	High physiological stability during establishment
**BS**	T0–T9	High but heterogeneous	Variable among treatments; progressive in some lines	Variable	Marked physiological heterogeneity among experimental lines
**H**	T0–T9	High in numerous lines	Mainly peripheral; progressive in some combinations	Partial in the most affected lines	Good initial preservation with localized deterioration
**T**	T0–T9	Variable among treatments	Frequently progressive	More pronounced in specific lines	Heterogeneous physiological response during establishment
**AA**	T0–T6	Maintained during the evaluable period	Variable	Not consistently quantifiable	Tissue remained evaluable during early culture; subsequent fate was incompletely documented for some lines
**BB**	T0–T6	Maintained during the evaluable period	Variable	Not consistently quantifiable	Heterogeneous physiological behavior among lines
**P**	T0–T6	Variable among lines	Low to moderate	Variable	Tissue persistence depended on the experimental combination
**R**	T0–T9	High	Generally low	Limited during the early period	High structural stability during early establishment

Note. Oxidation and necrosis were evaluated independently from tissue viability. The absence of subsequent observations was not interpreted as mortality when longitudinal continuity of the corresponding experimental line could not be unequivocally established. Treatment ranges correspond to the experimental design used for each explant type. Physiological responses are presented descriptively and should not be interpreted as independent quantitative estimates of survival.

Table S2. Temporal dynamics and morphological characteristics of callus development in *Hippeastrum × hybridum* explants.

EXPLANT	FIRST DETECTABLE PROLIFERATION	FIRST CLEARLY DIFFERENTIATED CALLUS	TREATMENTS/LINES SHOWING THE MOST RELEVANT CALLOGENIC RESPONSE	MAIN MORPHOLOGICAL OBSERVATIONS
**H**	~W3 (~21 d)	Mainly from ~W8 (~56 d)	T2 and T3 showed early proliferation; T5 showed abundant initial growth; T8 displayed high persistence at later stages; T4 also maintained persistent callus	Callus predominantly cream–yellow to greenish depending on the line. Early-responsive lines frequently developed progressive oxidation, whereas some later-persisting lines showed lower relative deterioration
**BS**	Proliferative changes preceded clear callus formation; evident before ~W8	Mainly from ~W8 (~56 d)	T5 produced abundant callus; T4 showed persistent tissue with comparatively low oxidation; T6 displayed an intermediate response	Callus ranged from cream–yellow to orange and was predominantly nodular, granular, or heterogeneous. Oxidation increased in some highly proliferative lines during prolonged culture
**B1**	~W2 (~14 d)	From ~W4 (~28 d)	T1 and T9 showed high callogenesis frequencies; T8 was notable for persistence and apparent callus accumulation; T7 combined callogenic and organogenic responses	Callus predominantly cream to yellow, with compact, nodular, or granular morphology depending on the line
**B2**	~W2–W3 (~14–21 d)	Mainly ~W5–W8 (~35–56 d)	T5 and T8 showed the most pronounced and persistent callogenic responses	Callus generally cream–yellow, with nodular, compact, or granular morphology. Callogenesis and organogenesis showed different treatment-dependent patterns
**T**	Proliferative changes occurred before clear callus formation, mainly before ~W8	Mainly ~W8 (~56 d); a markedly delayed response was observed in T8 at ~W24 (~168 d)	T2 and T3 showed abundant early proliferation; T6 maintained callus more persistently at later stages; T8 showed a delayed response	Early proliferative callus frequently showed progressive oxidation. Persistent callus was observed in selected lines, but no subsequent organogenic response developed
**AA**	Not observed	Not observed	—	Tissue partially retained its original structure during the early culture period, with variable oxidation but no confirmed callogenic development
**BB**	Not observed	Not observed	—	No callogenic response was observed. Tissue persistence and oxidation varied among lines during the evaluable period
**P**	Not observed	Not observed	—	Variable tissue persistence and coloration were observed, but no differentiated callus masses developed
**R**	Not observed	Not observed	—	Root segments largely retained their original morphology and generally showed low oxidation during the early culture period, without callogenic or regenerative development

Note. W indicates experimental week (W1 ≈ 7 days). First detectable proliferation refers to newly proliferating tissue observed before the formation of an unequivocally differentiated callus mass. Reported times are approximate and correspond to the first interval in which the respective response could be morphologically confirmed. Apparent callus accumulation was assessed descriptively and does not represent a gravimetric measurement of fresh or dry biomass. Material generated through subsequent fragmentation or subculture remained associated with its original experimental line and was not considered an independent biological replicate.

Table S3. Micropropagation and bulbification responses of advanced regenerative lines of *Hippeastrum × hybridum*

EXPLANT	ADVANCED SERIES EVALUATED (N)	SERIES WITH ROOTS AND/OR ORGANIZED GROWTH (N)	SERIES WITH CONFIRMED BULBIFICATION, N/N (%)	TREATMENTS WITH CONFIRMED BULBIFICATION	MINIMUM VISIBLE BULBIFICATION IN POSITIVE SERIES	MAIN RESPONSE CHARACTERISTICS
**B1**	11	8	**8/11 (72.7)**	T0, T1, T2, T7, T9	1–6 distinguishable bulbiform units per positive series	Highest continuity of multiplication. T7 showed the most pronounced bulbous proliferation, together with active root and leaf development
**B2**	6	6	**4/6 (66.7)**	T0, T1, **T7**, T9	≥1 bulbiform unit per positive series	Confirmed bulbification in T0, T1, T7, and T9. T2 showed marked regenerative and rooting activity but no unequivocal bulb differentiation
**BS**	8	3	**2/8 (25.0)**	T2, T7	T2: ≥2; T7: ≥5 distinguishable bulbiform units	More restricted bulbification. T7 showed the strongest response, with multiple bulbous units and abundant root and leaf development; T3 regenerated without unequivocal bulb formation

Note. B1, basal bulb fraction including the basal plate; B2, apical bulb fraction without the basal plate; BS, ovary. Percentages were calculated relative to the number of advanced regenerative series evaluated and therefore do not represent regeneration frequencies from the initial explants. Bulbiform-unit numbers indicate the minimum number of clearly distinguishable units observed within positive series and are presented descriptively. Because regenerated bulbous structures were subsequently fragmented during micropropagation, derived units were not considered independent biological replicates or used to calculate a multiplication rate.

Table S4. GC–MS-detected compounds and unidentified signals in cellular and extracellular alkaloid extracts of B1 and BS *Hippeastrum* × *hybridum* cell suspension cultures during coronatine elicitation.

Signal / alkaloid assignment	RT (min)	RI ref.	BS D0 Media	BS D0 Cells	BS D7 Media	BS D7 Cells	BS D14 Media	BS D14 Cells	B1 D0 Media	B1 D0 Cells	B1 D7 Media	B1 D7 Cells	B1 D14 Media	B1 D14 Cells
**Identified**			**0.000**	**0.000**	**0.000**	**0.000**	**0.000**	**0.000**	**0.000**	**0.000**	**20.472**	**0.000**	**0.000**	**0.000**
**Hordenine**	11.50	1469									4.267			
**O-Methylnorbelladine**	24.66	2602.3									16.205			
**Unidentified signals**			**16.516**	**0.000**	**52.733**	**10.277**	**12.930**	**7.400**	**0.000**	**0.000**	**37.878**	**51.169**	**46.155**	**44.215**
**Unknown (m/z 107/55)**	14.80	1461.7						3.673						
**Unknown (m/z 124/77)**	16.80	2786				3.669								
**Unknown (m/z 189/161)**	19.04	2170.5	2.158											
**Unknown (m/z 240/78)**	21.30	2777.8											4.432	
**Unknown (m/z 128/57)**	24.36	2502.1	2.814											
**Unknown (m/z 57/137)**	25.00	2602.3										6.520		
**Unknown (m/z 259/57)**	25.69	2589.8	2.745											
**Unknown (m/z 134/135)**	25.90	1461.7												4.705
**Unknown (m/z 270/166)**	26.90	2918.9									4.156			
**Unknown (m/z 273/57)**	27.14	2688	3.224											
**Unknown (m/z 166/270)**	27.30	2918.9										6.418	4.514	7.246
**Unknown (m/z 207/55)**	27.40	1461.7									3.999			
**Unknown (m/z 129/57)**	27.60	2372.8			8.599	6.608						7.672		
**Unknown (m/z 259/273)**	27.94	2744.2	3.217											
**Unknown (m/z 152/284)**	28.60	2483												5.986
**Unknown (m/z 91/207)**	28.80	2627											12.494	
**Unknown (m/z 149/57)**	29.10	2348.6					12.930							
**Unknown (m/z 149/167)**	29.40	2348.6			24.808							23.758	9.209	

**Unknown (m/z 207/91)**	29.40	2672									4.157		7.635	
**Unknown (m/z 284/256)**	30.10	2483												4.844
**Unknown (m/z 207/281)**	30.50	2964.3									4.310			
**Unknown (m/z 137/207)**	31.30	2602.3									5.430			
**Unknown (m/z 207/57)**	31.36	2994.3	2.358											
**Unknown (m/z 286/166)**	31.50	2413												10.095
**Unknown (m/z 180/300)**	32.00	1699.2												5.390
**Unknown (m/z 150/272)**	32.10	2641.3			19.326			3.728			9.474	6.802	7.871	
**Unknown (m/z 207/300)**	32.90	2602.3									6.352			
**summed quantified signal**			**16.516**	**0.000**	**52.733**	**10.277**	**12.930**	**7.400**	**0.000**	**0.000**	**58.350**	**51.169**	**46.155**	**44.215**

Note. D0 corresponds to sampling immediately before the addition of 1 μM coronatine (COR), whereas D7 and D14 correspond to 7 and 14 days after elicitation, respectively. Values are expressed as μg galantamine equivalents per mg of alkaloid extract (μg Gal-eq·mg^{-1}). For compounds other than galantamine, values represent relative estimates based on the galantamine calibration curve and should not be interpreted as absolute concentrations. Blank cells indicate features that were not detected or could not be reliably quantified under the applied analytical and data-processing conditions.

Table S5. GC–MS-detected structurally assigned compounds and unidentified features in *in vitro* and *ex vitro* tissues of *Hippeastrum* × *hybridum*.

Signal / alkaloid assignment	RT (min)	RI exp.	R- invitro	B- invitro	H- invitro	B-exvitro	H-exvitro
**Homolycorine-type**			**2.223**	**2.389**	**2.79**	**0**	**2.685**
**Candimine**	33.08	3127.8	2.223	2.389	2.790		2.685
**Unidentified signals**			**4.163**	**8.633**	**15.185**	**16.964**	**9.497**
**Unknown (m/z 189/161)**	19.04	2170.2			2.079	2.058	2.040
**Unknown (m/z 273/259)**	22.86	2405.4			2.440		
**Unknown (m/z 245/259)**	24.22	2493				2.405	
**Unknown (m/z 270/329)**	24.45	2507.8					
**Unknown (m/z 259/273)**	25.67	2588.9		2.085		2.590	
**Unknown (m/z 57/273)**	25.79	2596.7		2.202			
**Unknown (m/z 287/105)**	25.79	2596.7			2.538		
**Unknown (m/z 57/301)**	25.81	2597.8	2.112				
**Unknown (m/z 287/57)**	25.81	2597.8					2.672
**Unknown (m/z 273/259)**	27.12	2686.9		2.221	2.976	2.744	
**Unknown (m/z 287/301)**	27.20	2692.6			2.221		
**Unknown (m/z 259/273)**	27.93	2743.8	2.051	2.125	2.931	2.679	2.615
**Unknown (m/z 127/57)**	30.32	2916.4				2.365	
**Unknown (m/z 273/287)**	30.66	2941.5				2.123	2.170
**summed quantified signal**			**6.386**	**11.022**	**17.975**	**16.964**	**12.182**

Note. Alkaloid extracts from *in vitro* roots (R), bulbs (B), and leaves (H), and from *ex vitro* bulbs and leaves were analyzed by GC–MS. Values are expressed as μg galantamine equivalents per mg of alkaloid extract (μg Gal-eq·mg^{-1}). Structural assignments were based on retention time, experimental retention index (RI), mass spectral characteristics, comparison with the specialized Amaryllidaceae alkaloid library, and available reference data. Signals that could not be assigned to a specific compound were reported as unidentified. Grouped values for phenanthridine-, narciclasine-, and homolycorine-type signals correspond to the summed contribution of compounds assigned to each structural class. Blank cells indicate features that were not detected or could not be reliably quantified under the applied analytical and data-processing conditions. Values for compounds other than galantamine represent relative estimates based on the galantamine calibration curve and should not be interpreted as absolute concentrations.

Table S6. Treatment-specific callogenic and organogenic responses of *Hippeastrum* × *hybridum* explants subjected to different plant growth regulator treatments.

Explant (code)	Treatment	Initial explants (N)	Callogenesis, n/N (%)	Root formation, n/N (%)	Shoot formation, n/N (%)	Leaf formation, n/N (%)	Overall organogenesis, n/N (%)
Leaf (H)	T1	20	15/20 (75.0)	0/20 (0.0)	0/20 (0.0)	0/20 (0.0)	0/20 (0.0)
	T2	20	17/20 (85.0)	0/20 (0.0)	0/20 (0.0)	0/20 (0.0)	0/20 (0.0)
	T3	20	20/20 (100.0)	0/20 (0.0)	0/20 (0.0)	0/20 (0.0)	0/20 (0.0)
	T4	20	12/20 (60.0)	0/20 (0.0)	0/20 (0.0)	0/20 (0.0)	0/20 (0.0)
	T5	20	19/20 (95.0)	0/20 (0.0)	0/20 (0.0)	0/20 (0.0)	0/20 (0.0)
	T6	20	9/20 (45.0)	0/20 (0.0)	0/20 (0.0)	0/20 (0.0)	0/20 (0.0)
	T7	20	16/20 (80.0)	1/20 (5.0)	0/20 (0.0)	0/20 (0.0)	1/20 (5.0)
	T8	20	14/20 (70.0)	0/20 (0.0)	0/20 (0.0)	0/20 (0.0)	0/20 (0.0)
Ovary (BS)	T1	20	15/20 (75.0)	0/20 (0.0)	0/20 (0.0)	0/20 (0.0)	0/20 (0.0)
	T2	20	17/20 (85.0)	1/20 (5.0)	3/20 (15.0)	3/20 (15.0)	3/20 (15.0)
	T3	20	16/20 (80.0)	2/20 (10.0)	5/20 (25.0)	6/20 (30.0)	6/20 (30.0)
	T4	20	13/20 (65.0)	0/20 (0.0)	0/20 (0.0)	0/20 (0.0)	0/20 (0.0)
	T5	20	17/20 (85.0)	0/20 (0.0)	0/20 (0.0)	0/20 (0.0)	0/20 (0.0)
	T6	20	12/20 (60.0)	0/20 (0.0)	0/20 (0.0)	0/20 (0.0)	0/20 (0.0)
Basal bulb fraction (B1)	T0	6	1/6 (16.7)	3/6 (50.0)	1/6 (16.7)	1/6 (16.7)	3/6 (50.0)
	T1	6	5/6 (83.3)	1/6 (16.7)	1/6 (16.7)	1/6 (16.7)	1/6 (16.7)
	T2	6	4/6 (66.7)	1/6 (16.7)	1/6 (16.7)	1/6 (16.7)	1/6 (16.7)
	T3	6	3/6 (50.0)	0/6 (0.0)	0/6 (0.0)	0/6 (0.0)	0/6 (0.0)
	T4	6	3/6 (50.0)	0/6 (0.0)	0/6 (0.0)	0/6 (0.0)	0/6 (0.0)
	T5	6	2/6 (33.3)	0/6 (0.0)	0/6 (0.0)	0/6 (0.0)	0/6 (0.0)
	T6	6	4/6 (66.7)	0/6 (0.0)	0/6 (0.0)	0/6 (0.0)	0/6 (0.0)
	T7	6	4/6 (66.7)	4/6 (66.7)	2/6 (33.3)	4/6 (66.7)	4/6 (66.7)
	T8	6	5/6 (83.3)	0/6 (0.0)	0/6 (0.0)	0/6 (0.0)	0/6 (0.0)
	T9	6	2/6 (33.3)	1/6 (16.7)	1/6 (16.7)	1/6 (16.7)	1/6 (16.7)
Floral scape (T)	T0	9	0/9 (0.0)	0/9 (0.0)	0/9 (0.0)	0/9 (0.0)	0/9 (0.0)
	T1	10	1/10 (10.0)	0/10 (0.0)	0/10 (0.0)	0/10 (0.0)	0/10 (0.0)

	T2	10	4/10 (40.0)	0/10 (0.0)	0/10 (0.0)	0/10 (0.0)	0/10 (0.0)
	T3	10	7/10 (70.0)	0/10 (0.0)	0/10 (0.0)	0/10 (0.0)	0/10 (0.0)
	T4	10	4/10 (40.0)	0/10 (0.0)	0/10 (0.0)	0/10 (0.0)	0/10 (0.0)
	T5	10	7/10 (70.0)	0/10 (0.0)	0/10 (0.0)	0/10 (0.0)	0/10 (0.0)
	T6	10	3/10 (30.0)	0/10 (0.0)	0/10 (0.0)	0/10 (0.0)	0/10 (0.0)
	T7	10	1/10 (10.0)	0/10 (0.0)	0/10 (0.0)	0/10 (0.0)	0/10 (0.0)
	T8	10	4/10 (40.0)	0/10 (0.0)	0/10 (0.0)	0/10 (0.0)	0/10 (0.0)
	T9	10	3/10 (30.0)	0/10 (0.0)	0/10 (0.0)	0/10 (0.0)	0/10 (0.0)
**Apical bulb fraction (B2)**	T0	8	1/8 (12.5)	1/8 (12.5)	2/8 (25.0)	2/8 (25.0)	2/8 (25.0)
	T1	8	5/8 (62.5)	1/8 (12.5)	1/8 (12.5)	1/8 (12.5)	1/8 (12.5)
	T2	8	3/8 (37.5)	2/8 (25.0)	2/8 (25.0)	2/8 (25.0)	2/8 (25.0)
	T3	8	2/8 (25.0)	0/8 (0.0)	0/8 (0.0)	0/8 (0.0)	0/8 (0.0)
	T4	8	2/8 (25.0)	0/8 (0.0)	0/8 (0.0)	0/8 (0.0)	0/8 (0.0)
	T5	8	3/8 (37.5)	0/8 (0.0)	0/8 (0.0)	0/8 (0.0)	0/8 (0.0)
	T6	8	2/8 (25.0)	0/8 (0.0)	0/8 (0.0)	0/8 (0.0)	0/8 (0.0)
	T7	8	2/8 (25.0)	0/8 (0.0)	0/8 (0.0)	0/8 (0.0)	0/8 (0.0)
	T8	8	3/8 (37.5)	1/8 (12.5)	0/8 (0.0)	0/8 (0.0)	1/8 (12.5)
	T9	8	3/8 (37.5)	1/8 (12.5)	1/8 (12.5)	1/8 (12.5)	1/8 (12.5)
**Stamen filaments from open flowers (AA)**	T1	20	0/20 (0.0)	0/20 (0.0)	0/20 (0.0)	0/20 (0.0)	0/20 (0.0)
	T2	20	0/20 (0.0)	0/20 (0.0)	0/20 (0.0)	0/20 (0.0)	0/20 (0.0)
	T3	20	0/20 (0.0)	0/20 (0.0)	0/20 (0.0)	0/20 (0.0)	0/20 (0.0)
	T4	20	0/20 (0.0)	0/20 (0.0)	0/20 (0.0)	0/20 (0.0)	0/20 (0.0)
	T5	20	0/20 (0.0)	0/20 (0.0)	0/20 (0.0)	0/20 (0.0)	0/20 (0.0)
	T6	10	0/10 (0.0)	0/10 (0.0)	0/10 (0.0)	0/10 (0.0)	0/10 (0.0)
**Stamen filaments from closed flower buds (BB)**	T1	20	0/20 (0.0)	0/20 (0.0)	0/20 (0.0)	0/20 (0.0)	0/20 (0.0)
	T2	20	0/20 (0.0)	0/20 (0.0)	0/20 (0.0)	0/20 (0.0)	0/20 (0.0)
	T3	20	0/20 (0.0)	0/20 (0.0)	0/20 (0.0)	0/20 (0.0)	0/20 (0.0)
	T4	20	0/20 (0.0)	0/20 (0.0)	0/20 (0.0)	0/20 (0.0)	0/20 (0.0)
	T5	20	0/20 (0.0)	0/20 (0.0)	0/20 (0.0)	0/20 (0.0)	0/20 (0.0)
	T6	20	0/20 (0.0)	0/20 (0.0)	0/20 (0.0)	0/20 (0.0)	0/20 (0.0)
**Isolated anthers (P)**	T1	20	0/20 (0.0)	0/20 (0.0)	0/20 (0.0)	0/20 (0.0)	0/20 (0.0)
	T2	20	0/20 (0.0)	0/20 (0.0)	0/20 (0.0)	0/20 (0.0)	0/20 (0.0)

	T3	20	0/20 (0.0)	0/20 (0.0)	0/20 (0.0)	0/20 (0.0)	0/20 (0.0)
	T4	20	0/20 (0.0)	0/20 (0.0)	0/20 (0.0)	0/20 (0.0)	0/20 (0.0)
	T5	20	0/20 (0.0)	0/20 (0.0)	0/20 (0.0)	0/20 (0.0)	0/20 (0.0)
	T6	18	0/18 (0.0)	0/18 (0.0)	0/18 (0.0)	0/18 (0.0)	0/18 (0.0)
**Root (R)**	T0	14	0/14 (0.0)	0/14 (0.0)	0/14 (0.0)	0/14 (0.0)	0/14 (0.0)
	T1	14	0/14 (0.0)	0/14 (0.0)	0/14 (0.0)	0/14 (0.0)	0/14 (0.0)
	T2	14	0/14 (0.0)	0/14 (0.0)	0/14 (0.0)	0/14 (0.0)	0/14 (0.0)
	T3	14	0/14 (0.0)	0/14 (0.0)	0/14 (0.0)	0/14 (0.0)	0/14 (0.0)
	T4	14	0/14 (0.0)	0/14 (0.0)	0/14 (0.0)	0/14 (0.0)	0/14 (0.0)
	T5	14	0/14 (0.0)	0/14 (0.0)	0/14 (0.0)	0/14 (0.0)	0/14 (0.0)
	T6	14	0/14 (0.0)	0/14 (0.0)	0/14 (0.0)	0/14 (0.0)	0/14 (0.0)
	T7	14	0/14 (0.0)	0/14 (0.0)	0/14 (0.0)	0/14 (0.0)	0/14 (0.0)
	T8	14	0/14 (0.0)	0/14 (0.0)	0/14 (0.0)	0/14 (0.0)	0/14 (0.0)
	T9	14	0/14 (0.0)	0/14 (0.0)	0/14 (0.0)	0/14 (0.0)	0/14 (0.0)

Note. Responses were calculated relative to the number of initially inoculated explants included in each explant–treatment combination. Callogenesis was recorded only when a clearly differentiated and persistent callus response was confirmed. Root, shoot, and leaf formation were recorded independently, whereas overall organogenesis represents the number of explants that developed at least one newly formed organized structure. Each explant was counted only once for overall organogenesis, irrespective of the number or type of structures produced. Responses arising subsequently from fragmentation, prolonged subculture, or multiplication of derived material were not considered independent initial response events. T0 corresponds to the control treatment without plant growth regulators.
